# Targeted *OUM1/PTPRZ*1 silencing and synergetic CDT/enhanced chemical therapy toward uveal melanoma based on a dual-modal imaging-guided manganese metal–organic framework nanoparticles

**DOI:** 10.1186/s12951-022-01643-y

**Published:** 2022-11-05

**Authors:** Yue Li, Fang Li, Hui Pan, Xiaolin Huang, Jie Yu, Xueru Liu, Qinghao Zhang, Caiwen Xiao, He Zhang, Leilei Zhang

**Affiliations:** 1 Department of Ophthalmology, Shanghai Ninth People’s Hospital Afflicted to Shanghai Jiao Tong University School of Medicine, Shanghai, 200011 China; 2 Shanghai Key Laboratory of Orbital Diseases and Ocular Oncology, Shanghai, 200011 China; 3 School of Life Science and Technology, Tongji University, Shanghai, 200092 China; 4 East China University of Science and Technology, Shanghai, 200237 China

**Keywords:** Uveal melanoma, LncRNA *OUM1*, Protein tyrosine phosphorylation, ICG-si*OUM1* + si*PTPRZ1* + Cis@MOF-PR, Fenton-like reaction, Magnetic resonance

## Abstract

Metastasis and chemical resistance are the most serious problems in the treatment of highly aggressive uveal melanoma (UM). The newly identified lncRNA *OUM1* is overexpressed in UM, functions as a catalyst and regulates protein tyrosine phosphatase (PTP) activity by binding to PTP receptor type Z1 (PTPRZ1), which plays an important role in cell proliferation, metastasis and chemotherapy resistance in the UM microenvironment. Hence, siRNAs that selectively knocking down the lncRNA *OUM1* (*siOUM1*) and its target gene *PTPRZ1* (*siPTPRZ1)* were designed to inhibit the *OUM1*/*PTPRZ1* pathway to reduce PTP activity, and this reduction in activity interrupts protein tyrosine phosphorylation, suppresses UM proliferation and metastasis and improves cisplatin sensitivity in UM cells. Then, to overcome the limitations of the difficulty of drug administration and traditional therapeutics, the indocyanine green (ICG)-labeled manganese metal–organic framework (MOF) nanoparticles (NPs) were fabricated and linked with arginine-glycine-aspartate (RGD) peptide to carry *siOUM1/siPTPRZ1* and cisplatin to achieve targeted siRNA interference-mediated therapy, enhanced cisplatin therapy and chemodynamic therapy. This NP system also has a dual-modal imaging ability because ICG is a near-infrared region fluorescent dye and manganese has the potential to be used in magnetic resonance imaging. This study verifies the significance of the newly discovered lncRNA *OUM1* as a new therapeutic target for aggressive UM and provides a drug delivery NP system for precise treatment of UM accompanied with a dual-modal imaging ability.

**Graphical Abstract:**

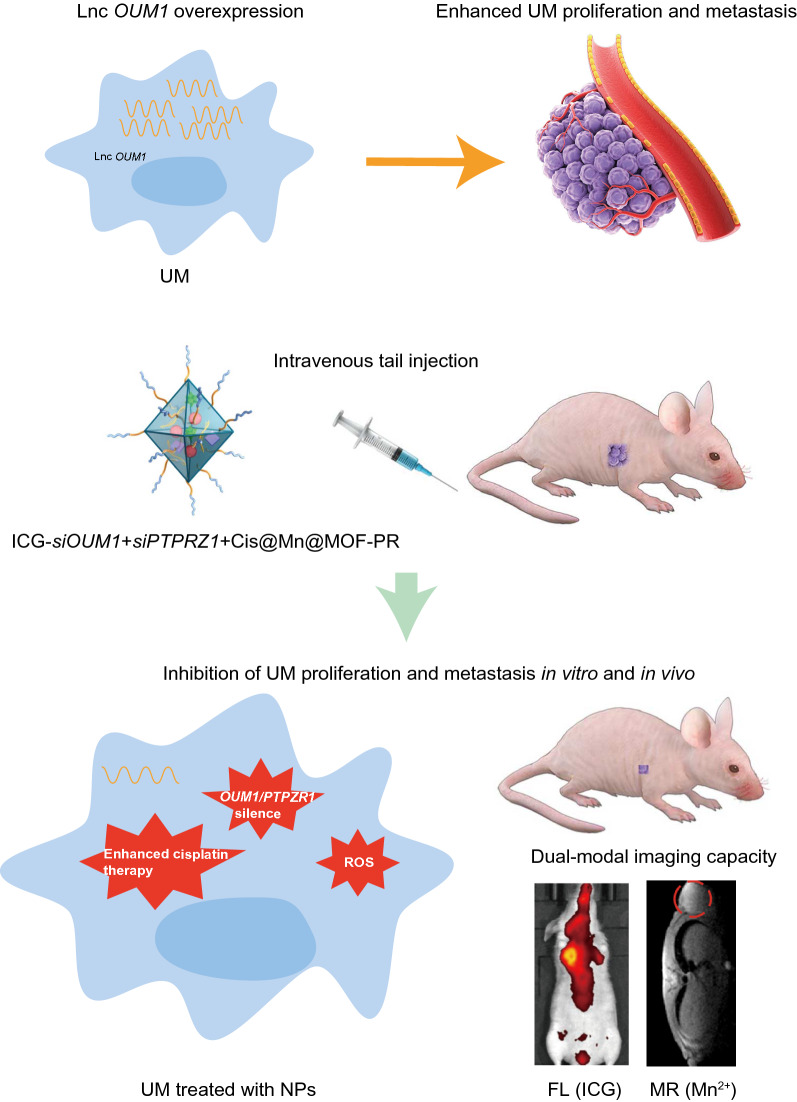

**Supplementary Information:**

The online version contains supplementary material available at 10.1186/s12951-022-01643-y.

## Introduction

Uveal melanoma (UM) is the most common and aggressive intraocular tumor in adults and shows a high mortality rate, with frequent recurrence and early regional lymph node involvement and subsequent metastasis [1–3]. The development and progression of melanoma have been attributed to genetic and epigenetic events, including methylation, chromatin modification and the diverse activities of noncoding RNAs [4]. Recently, the function of long noncoding RNAs (lncRNAs) in melanoma has gained increased attention. LncRNAs undergo typical mammalian RNA processing but do not have protein-coding capability [5, 6]; these RNA molecules exert a profound effect on the cellular transcriptional and translational landscape and the dysregulation of lncRNAs has been detected in various types of cancer [7–10]. In recent years, an increasing number of studies have focused on lncRNAs in UM.

Cancers develop in complex tissue environments and depend on these environments for sustained growth, invasion and metastasis [11]. Cytoplasmic lncRNAs are usually involved in the tumor microenvironment. Protein tyrosine phosphorylation plays a critical role in maintaining the tumor microenvironment. This process is governed by the balanced actions of protein tyrosine phosphatases (PTPs) responsible for dephosphorylation and protein tyrosine kinases (PTKs) that catalyze tyrosine phosphorylation [12]. The deregulation of phosphorylation is known to be involved in neoplastic disease [13, 14]. In this study, a novel cytoplasmic lncRNA, Oncotarget in UM Formation-Transcript 1 (*OUM1*), and its downstream target gene PTP receptor type Z1 (*PTPRZ1*) were detected in UM tissues and cells. We also identified the potential role of *OUM1* in regulating PTP activity in the tumor microenvironment through *PTPRZ1* and its effect on UM progression. Our study thus reveals a previously unappreciated regulatory mechanism of lncRNAs that regulates PTP activity through *PTPRZ1*. These findings broaden the known mechanisms of action of lncRNAs in UM and suggest that the inhibition of *OUM1* and *PTPRZ1* may provide a novel means for targeting UM.

However, although RNA interference-mediated therapy has been studied for the past 20 years, this therapy has suffered from a lack of effective oligonucleotide-delivery systems [15]. In addition, traditional intravenous chemotherapy lacks specificity, and chemotherapy drugs also produce toxic and adverse side effects on normal tissues [16]. In recent years, drug delivery nanoparticles (NPs) have been developed rapidly to overcome the limitations of traditional therapeutics and navigate biological barriers to realize more targeted therapy [17]. Due to their porosity, diverse chemical functionalities and well-defined, 3D architecture, metal–organic framework NPs have shown great superiority in a wide range of areas, including drug delivery, and serve as biological probes [18–20]. Arginine-glycine-aspartate (RGD) peptide is an extraordinary targeting ligand toward tumors and due to its specific interaction with integrin αvβ3 receptors, which are overexpressed on diverse tumor cell membranes and the related neovasculature, RGD has been applied to the modification of various biomimetic interfaces to realize specific targeting ability [21–23]. Polyethylene glycol (PEG), which is devoid of any steric hindrance and is known for its high structural flexibility, amphiphilicity, biocompatibility and high hydration capacity, could improve the drug targeting and bioavailability of NPs [24, 25]. Studies have shown that the pH value of the microenvironment of solid tumor tissue is approximately 6.5 with a range of 5.66–7.78 [26, 27], which is lower than that of normal tissue (pH 7.4). Iron-based nanomaterials can dissolve ferrous ions under the mildly acidic conditions of the tumor microenvironment and initiate the Fenton reaction to overproduce H_2_O_2_, which results in the generation of reactive oxygen species (ROS), ·OH to trigger apoptosis and to inhibit tumors; this newly defined therapeutic strategy is chemodynamic therapy (CDT) [28–30]. Metal–organic framework NPs will degrade in the acidic microenvironment of solid tumors and release loaded drug [31, 32]. Through a judicious choice of inorganic and organic components, the chemical functionalities and crystalline structure of metal–organic frameworks can be deliberately modulated to be utilized for CDT and to function as a siRNA delivery system [33].

Herein, we designed a pH-sensitive drug delivery NP system linked with PEG and RGD sequences. *SiOUM1, siPTPRZ1* and cisplatin were loaded into the pores of indocyanine green (ICG) labeled Mn@MOF. ICG is a near-infrared region (NIR) dye that has been used in clinical application and exhibits excellent biocompatibility [34]. This dye can also serve as an NIR fluorescence probe that allows detection via confocal laser scanning microscopy (CLSM) or an in vivo imaging system (IVIS). *SiOUM1* and *siPTPRZ1* inhibit the *OUM1/PTPRZ1* pathway and thus suppress UM proliferation and metastasis and improve the cisplatin sensitivity of UM cells. Mn^2+^ can induce a Fenton-like reaction and also enable detection via magnetic resonance imaging (MRI) [35]. With the exception of its dual imaging ability, this NP system with theranostic capacity is expected to achieve synergistic antitumor efficacy through a combination of precise RNA interference-mediated therapy, enhanced chemical therapy and CDT. Moreover, the magnetic NPs, which could be visualized by MRI in our study, support the promise of future clinical translation.

## Results and discussion

### *OUM1* is overexpressed in melanoma tissues and UM cells, and the knockdown of *OUM1* can inhibit cell proliferation, invasion and xenograft growth

**Fig. 1:**
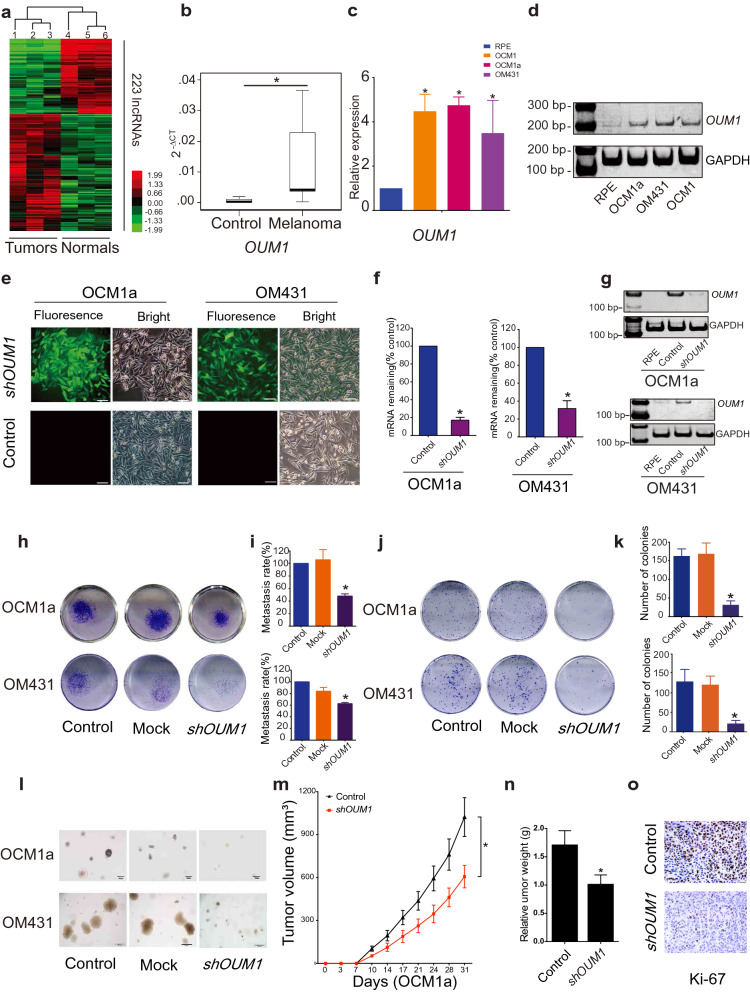
*OUM1* overexpressed in melanoma tissues and UM cells and *OUM1* knockdown inhibited cell proliferation and invasion. **a** Hierarchical clustering analysis of 223 lncRNAs that were differentially expressed between melanoma samples and nontumor samples. **b** The lncRNA *OUM1* was significantly overexpressed in melanoma tissues compared with normal tissues. **c** *OUM1* showed higher expression in the melanoma cell lines OCM1, OCM1a and OM431 than in RPE cells (negative control), as measured by qRT-PCR. **d** *OUM1* expression was measured by RT-PCR in different UM and normal cells. Triplicate assays were performed and the relative level of *OUM1* was normalized to GAPDH (*P < 0.05). **e** *OUM1* was knocked down by one shRNA. EGFP was applied to track *OUM1* shRNA expression. **f**, **g** *OUM1* was downregulated in UM cells, as shown by qRT-PCR and RT-PCR. **h** Images of the Transwell assays. **i** Migrated cells that were fixed with 5% paraformaldehyde, stained with 0.1% crystal violet and washed with 33% acetic acid were quantified. The absorbance of the collected liquid was measured at 570 nm with a microplate reader. **j** Images of colony formation assays. **k** Colonies were fixed, stained, washed and measured with a microplate reader at 570 nm. **l** Images of colonies in soft agar. Few small colonies were observed in the *OUM1* knockdown group. **m** Nu/Nu nude mice were injected with OCM1a or *shOUM1*-OCM1a cells. Tumor volume was measured twice a week, every 3 or 4 days. The xenografts grew slowly in mice injected with *shOUM1*-OCM1a cells compared to those injected with control cells (n = 6 mice per group). **n** After 31 days, the mice were sacrificed, and the tumors were removed and analyzed. The tumors from mice injected with *shOUM1*-OCM1a cells weighted less. **o** Ki-67 expression was evaluated by IHC staining in mouse tumor tissues. Scale bar: 20 μm. Mock: empty pGIPZ vector; *P < 0.05 compared with the control

To investigate lncRNA expression in melanoma, a microarray containing probes targeting 12,784 lncRNAs was used, and more than 200 lncRNAs were found to be significantly differentially expressed (P < 0.05; fold change > 2) in three pairs of eyelid melanoma and normal tissue. Hierarchical clustering showed systematic variations in lncRNA expression between melanoma and normal tissues (Fig. 1a). Information from the National Center for Biotechnology Information (NCBI) for *LOC100505912* (Additional file 1: Fig. S1a), which was significantly upregulated in melanoma tissues (Fig. 1b) and UM cells (Fig. 1c, d), is provided. As detailed in UCSC and NCBI databases, *LOC100505912* has a length of 898 bp, contains 4 exons and is located at 4p15.2 (Additional file 1: Fig. S1b, yellow box). By rapid amplification of cDNA ends (RACE), we identified a novel 1,464 bp transcript spanning 7 exons in UM cells (Additional file 1: Fig. S1b, blue box). Using the GENCODE annotation of the human genome, we then confirmed the absence of coding evidence for this novel transcript. Collectively, these data show that this novel isoform of *LOC100505912* is a noncoding transcript in UM and a potential new oncoRNA, and we therefore named it Oncotarget in UM Formation-Transcript 1 (*OUM1*).

Subsequently, three siRNAs were designed for the knockdown of *OUM1* expression (Additional file 1: Fig. S1b-e). The siRNA-mediated knockdown of *OUM1* significantly decreased cell viability and cell migration (Additional file 1: Fig. S1f, g). In addition, we found high efficiency S2 splice sites in exon 7 of *OUM1*, which were selected to construct the pGIPZ *OUM1* shRNA plasmid. The pGIPZ shRNA vector harboring EGFP was packaged into lentiviruses and transduced into human OCM1a and OM431 cells. We observed green fluorescence in the transduced cells (Fig. 1e). We selected cell clones using puromycin and detected *OUM1* expression; *OUM1* expression was knocked down in *shOUM1*-OCM1a and *shOUM1*-OM431 cells (Fig. 1f, g). The 24-well transwell experiment indicated that the metastasis rate was decreased by 50% in *shOUM1*-OCM1a cells and by approximately 40% in *shOUM1*-OM431 cells, and both of these rates were markedly lower than those of the untreated OCM1a and OM431 cells (Fig. 1h, i). Subsequently, decreased colony numbers were observed in *shOUM*1-OCM1a cells and *shOUM1*-OM431 cells by the colony formation assay (Fig. 1j, k). In addition, the soft agar assay displayed fewer and smaller colonies in *shOUM*1-OCM1a cells and *shOUM1*-OM431 cells (Fig. 1l). These in vitro data indicated that *OUM1* promoted UM progression. In vivo experiments using nude mice were subsequently performed. Untreated OCM1a and the *shOUM1*-OCM1a cells were separately injected into nude mice (each group consisted of six mice). The tumor volume was measured once every 3–4 days (twice a week). The tumors grew more slowly in mice injected with *shOUM1*-OCM1a cells than in mice injected with untreated OCM1a cells (Fig. 1m). After 31 days, the mice were sacrificed, and the tumors were removed and analyzed. The tumor weights were lower in the *shOUM1*-OCM1a group (Fig. 1n). As observed by IHC staining, less Ki-67 expression was found in mouse tumor tissue of the *shOUM1*-OCM1a group at the end of the experiments (Fig. 1o). These data indicated that the proliferation and metastasis rates were impaired after *OUM1* knockdown in vivo and in vitro*. OUM1* might serve as a new oncoRNA in UM.

### *OUM1* contributes to tumor progression through its downstream target *PTPRZ1*

To investigate the role of *OUM1* in UM, a genome-wide cDNA array was employed to determine factors after *OUM1* knockdown. Compared with the expression in untreated OCM1a cells, *OUM1* knockdown downregulated and upregulated the expression of 86 and 63 genes, respectively (Additional file 1: Table S1). The molecular function analysis revealed that nucleotide binding, calcium ion binding, protein homodimerization activity, GTPase activity, integrin binding, growth factor activity, and extracellular matrix binding were significantly changed after *OUM1* knockdown. Nucleotide binding accounted for the largest proportion, which was approximately 32.1% (Additional file 1: Fig. S2). Bioformational analysis further verified that nucleotide-binding activity was associated with transmembrane receptor protein phosphatase, which was closely related to PTP activity (Additional file 1: Fig. S3). Among the differentially expressed genes after *OUM1* knockdown, only one gene, *PTPRZ1*, was associated with PTPs. The expression of *PTPRZ1* was significantly downregulated by approximately 3.9-fold (Additional file 1: Table S1). Other enzyme activities associated with genes were not detected; that is, *PTPRZ1* might act as a target of *OUM1* and might function as an oncogene in UM cells.

**Fig. 2:**
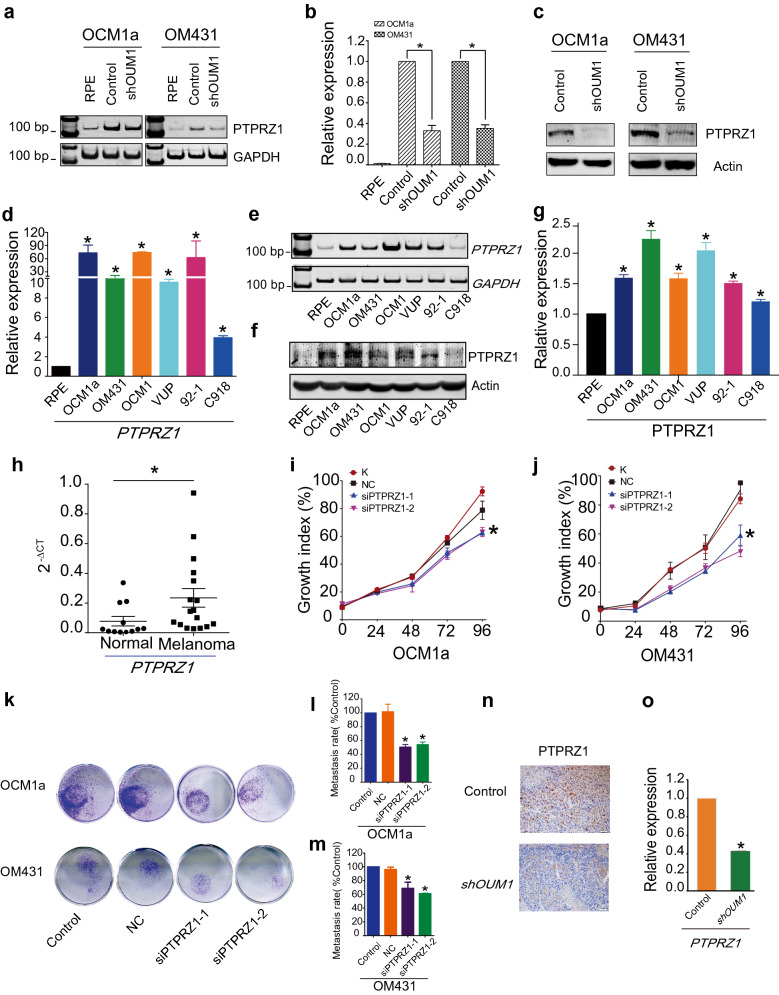
*OUM1* contributes to tumor progression through its downstream target *PTPRZ1.* **a**, **b** The mRNA expression of *PTPRZ1* was upregulated in untreated UM cells but downregulated after *OUM1* knockdown, as detected using qRT-PCR and native PAGE in OCM1a, OM431, *shOUM1*-OCM1a and *shOUM1*-OM431 cells. **c** The protein expression of PTPRZ1 decreased, as determined by western blot. **d**, **e** *PTPRZ1* expression was higher in a panel of UM cell lines (OCM1, OCM1a, OM431, VUP, 92–1 and C918) than in control RPE cells as measured by qRT-PCR and RT-PCR. **f**, **g** PTPRZ1 expression was higher in a panel of UM cell lines than in normal RPE cells as shown by western blot. **h** In melanoma tissues, PTPRZ1 expression increased significantly, as detected by qRT-PCR. *P < 0.05 compared with the control. **i**, **j** Cell proliferation was significantly decreased in OCM1a and OM431 cells after *PTPRZ1* silencing. **k** Images of metastatic *PTPRZ1*-silenced tumor cells. The migration assay was conducted 48 h after transfection with *siPTPRZ1* or control siRNA. **l**, **m** Quantitative analysis of metastatic tumor cells. The migratory ability of *siPTPRZ1*-treated OCM1a and OM431 tumor cells was significantly decreased. **n** IHC staining confirmed that PTPRZ1 protein expression was decreased in mouse tumors formed by *shOUM1*-OCM1a cells. **o** *PTPRZ1* expression was decreased in mouse tumors formed by *shOUM1*-OCM1a cells, as shown by qRT-PCR. *P < 0.05 compared with the control

We also demonstrated that *PTPRZ1* expression was decreased at both the mRNA level and protein levels after *OUM1* knockdown (Fig. 2a–c), whereas abundant *PTPRZ1* expression was detected at both the mRNA level and protein level in untreated UM cells (Fig. 2d–g). To verify the clinical significance of *PTPRZ1*, we examined whether *PTPRZ1* expression is correlated with *OUM1* expression in tumor tissues. *PTPRZ1* was abundantly expressed in tumor tissues (Fig. 2h) according to the expression of *OUM1*. Thus, *PTPRZ1* might be a target of *OUM1* and function as an oncoRNA in UM cells. We then knocked down *PTPRZ1* using two different siRNAs (*siPTPRZ1-1* and *siPTPRZ1-2*) designed to silence *PTPRZ1* expression in OCM1a and OM431 cells. As expected, *PTPRZ1* was successfully silenced by *siPTPRZ1-1* and *siPTPRZ1-2* (Additional file 1: Fig. S4a, b). Intriguingly, *OUM1* expression was not significantly changed in UM cells after the silencing of *PTPRZ1* (Additional file 1: Fig. S4c). To exclude off-target effects, we also investigated two downregulated genes, *FXYD3* and *ST8SIA1*. However, there were no significant changes in *FXYD3* or *ST8SIA1* expression were found in *shOUM1*-OCM1a and *shOUM1*-OM431 cells or in melanoma samples (Additional file 1: Fig. S4d-g). These data further confirm that *PTPRZ1* is the downstream regulatory target of *OUM1*.

Subsequently, the regulatory role of *PTPRZ1* in tumorigenesis was elucidated. Through MTT assays, we observed a decreased proliferation rate in OCM1a and OM431 cells after the silencing of *PTPRZ1* expression (Fig. 2i, j). Classical transwell assays showed a significantly decreased metastasis rate, which was consistent with the results after *OUM1* knockdown (Fig. 2k–m). We also detected *PTPRZ1* expression in tumor tissues from Nu/Nu nude mice injected with OCM1a or *shOUM1*-OCM1a cells. In a nude mouse xenograft model formed by *shOUM1*-OCM1a cells, decreased *PTPRZ1* expression was detected by immunohistochemistry (IHC) staining and quantitative reverse transcription-polymerase chain reaction (qRT-PCR) (Fig. 2n, o).

### *OUM1* is mainly localized in the cytoplasm and directly binds to the PTPRZ1 protein to enhance PTP activity

**Fig. 3:**
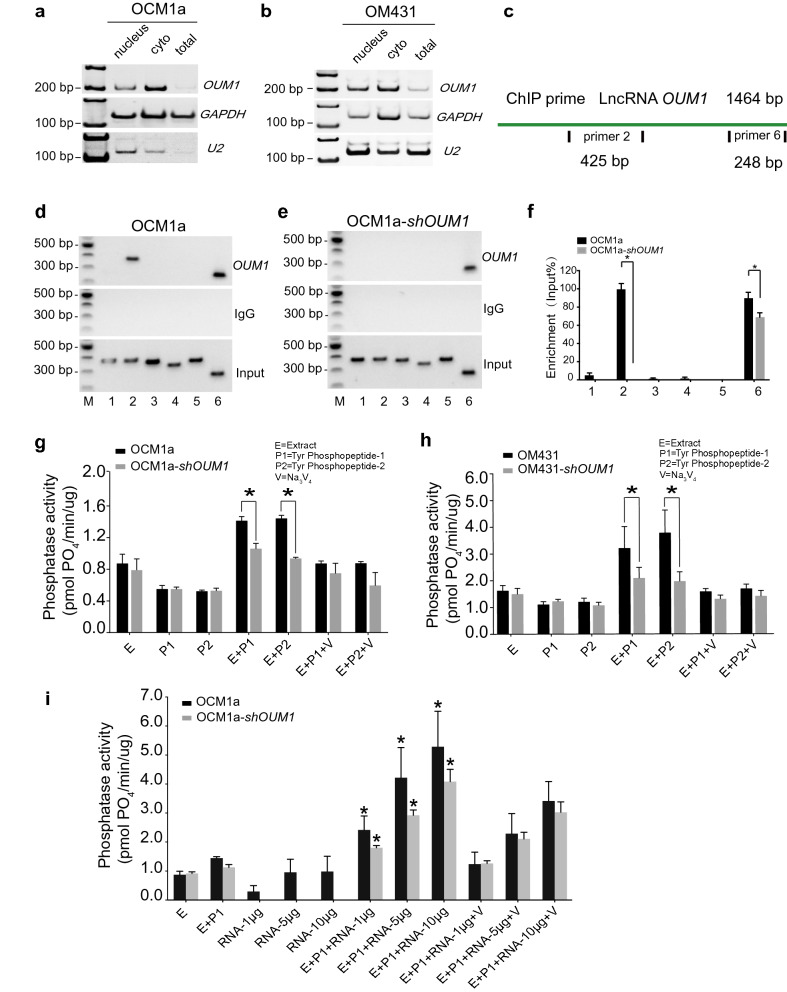
*OUM1* mainly locates in cytoplasm and directly binds to PTPRZ1 protein to enhance PTP activity. **a**, **b** *OUM1* mainly located in the cytoplasm in OCM1a and OM431 cells. U2 RNA: positive control for nuclear RNA. **c** Schematic of the regions responsible for binding between *OUM1* and PTPRZ1. Sites 2 and 6: different detection locations for RNA ChIP. **d**, **e** The interaction of *OUM1* and PTPRZ1 was detected. After *OUM1* knockdown, the binding between PTPRZ1 and *OUM1* was abolished at the primer 2 region and decreased at the primer 6 region in OCM1a-*shOUM1* cells. Input: total RNA reverse transcribed and amplified without incubation with the PTPRZ1 antibody. IgG: negative control. **f** Gray level analysis conducted to quantify the enrichment of *OUM1* on PTPRZ1. **g**, **h** PTP activity was detected in OCM1a and OM431 cells. The amount of phosphate indicates phosphatase activity. The amount of phosphate decreased from 1.4 pmol _PO4/min/μg_ and 1.5 pmol _PO4/min/μg_ to 1.0 pmol _PO4/min/μg_ and 0.9 pmol _PO4/min/μg_ when P1 and P2 were added, respectively, after *OUM1* knockdown in OCM1a cells. In OM431 cells, these values decreased from 3.2 pmol _PO4/min/μg_ and 3.7 pmol _PO4/min/μg_ to 2.1 pmol _PO4/min/μg_ and 2.0 pmol _PO4/min/μg_ when P1 and P2 were added, respectively, after *OUM1* knockdown. **i** Phosphate content increased significantly after synthesized *OUM1* RNA was added to the PTP reaction system at increasing concentrations of 1 μg, 5 μg and 10 μg; at these concentrations, the phosphate content was approximately 2.6 pmol _PO4/min/μg_, 4.2 pmol _PO4/min/μg_, and 5.2 pmol _PO4/min/μg_, respectively, in OCM1a cells and 1.8 pmol _PO4/min/μg_, 2.9 pmol _PO4/min/μg_, and 4.0 pmol _PO4/min/μg_, respectively, in *shOUM1*-OCM1a cells. *E* Extract, protein extracted from OCM1a, OM431, *shOUM1*-OCM1a or *shOUM1*-OM431 cells. P1 and P2 indicate two chemically synthesized phosphopeptides, Tyr phosphopeptides 1 (END(pY)INASL) and 2 (DADE(pY)LIPQQG). SOV (V): Na_3_VO_4_, a PTP inhibitor that inhibits PTP activity and is used as positive control. *P < 0.05

To gain insight into the underlying mechanism through which *OUM1* regulates *PTPRZ1* expression in UM, we examined the subcellular location of *OUM1*. By isolating nuclear, cytoplasmic and total RNA, *OUM1* was demonstrated to be mainly localized in the cytoplasm in OCM1a and OM431 cells (Fig. 3a, b). U2 snRNA was used as a positive reference [36].

Most studies have focused on lncRNAs localized in the nucleus and on their function through interactions with gene promoters or competition with a variety of histones; fewer studies have investigated cytoplasmic lncRNAs [37]. Therefore, the mechanism of the lncRNA *OUM1* was further explored. The RNA-chromatin immunoprecipitation (RNA-ChIP) assay confirmed that *OUM1* functions based on binding to the PTPRZ1 protein. Two binding sites for *OUM1* on PTPRZ1 were detected with primer 2 and primer 6 (Fig. 3c). In *shOUM1*-OCM1a cells, the primer 2 binding site between *OUM1* and PTPRZ1 was almost completely deleted, whereas that of primer 6 was impaired (Fig. 3d–f). This finding demonstrates that *OUM1* can function by directly binding to the PTPRZ1 protein in the cytoplasm.

We subsequently detected PTP activity based on the phosphate content of OCM1a and OM431 cells (Fig. 3g, h). Sodium orthovanadate (SOV, V) is a PTP inhibitor that has been employed as a negative control. In our study, PTP activity was activated only in the presence of both cellular protein extract (E) and substrate (two chemically synthesized phosphopeptides, P1: END(pY)INASL and P2: DADE(pY)LIPQQG). When the reactions contained only E, P1 or P2, the amount of phosphate was almost unchanged. After both E and P1 or P2 were added, the enzymes were significantly activated, and more phosphate was produced in OCM1a and OM431 cells, which overexpress *OUM1*. However, less phosphate was produced after *OUM1* knockdown. In addition, after the addition of different concentrations of *OUM1* RNA to the PTP reaction system, which was amplified, confirmed and purified, we discovered that the phosphate content was significantly increased in a dose-dependent manner (Fig. 3i). However, when *OUM1* RNA alone was added, the amount of phosphate formed was as low as that obtained when only E was added. A previous study confirmed that *OUM1* could directly bind to the *PTPRZ1* protein. We found that the novel cytoplasmic lncRNA *OUM1* acts as a catalyst by binding to PTPRZ1 to enhance PTP activity in UM cells and that the inhibition of *OUM1* could inhibit the proliferation and metastasis of UM in vivo and in vitro by interrupting protein tyrosine phosphorylation in the UM microenvironment. Given the strong upregulation of both *OUM1* and *PTPRZ1* in human malignant melanoma tissues and the finding that *OUM1* knockdown inhibits the expression of *OUM1* and subsequently decreases *PTPRZ1* activity, targeting *OUM1* and the downstream *PTPRZ1* may be a promising strategy to inhibit the proliferation and metastasis of UM.

SOV has shown promising antineoplastic activity in several human cancers [38, 39]. However, the effects of SOV on UM are relatively unknown. In this study, we detected the biological effect of UM cells after incubation with different concentrations of SOV. A MTT assay determined that the cell viability of OCM1a, *shOUM1*-OCM1a, OM431, *shOUM1*-OM431, MUM2b, OCM1, VUP and SP6.5 cells was suppressed in a dose-dependent manner. Incubation with increasing concentrations of SOV (10 μM, 25 μM, 50 μM and 100 μM) for 24 h, 48 h, 72 h and 96 h significantly suppressed the viability of UM cells (Additional file 1: Fig. S5a-h); that is, the survival rate was significantly lower in *shOUM1*-OCM1a cells (Additional file 1: Fig. S5e, f) and *shOUM1*-OM431 cells (Additional file 1: Fig. S5g, h) than in the control groups. These data suggest that lncRNA *OUM1* knockdown could enhance the antitumor activity of SOV and improve drug tolerance. This finding indicates that the application of chemical drugs such as cisplatin when *OUM1* and *PTPRZ1* are selectively knocked down and the *OUM1*/*PTPRZ1* pathway is inhibited could greatly improve drug tolerance and increase antitumor efficacy.

### Characterization of NPs

**Fig. 4:**
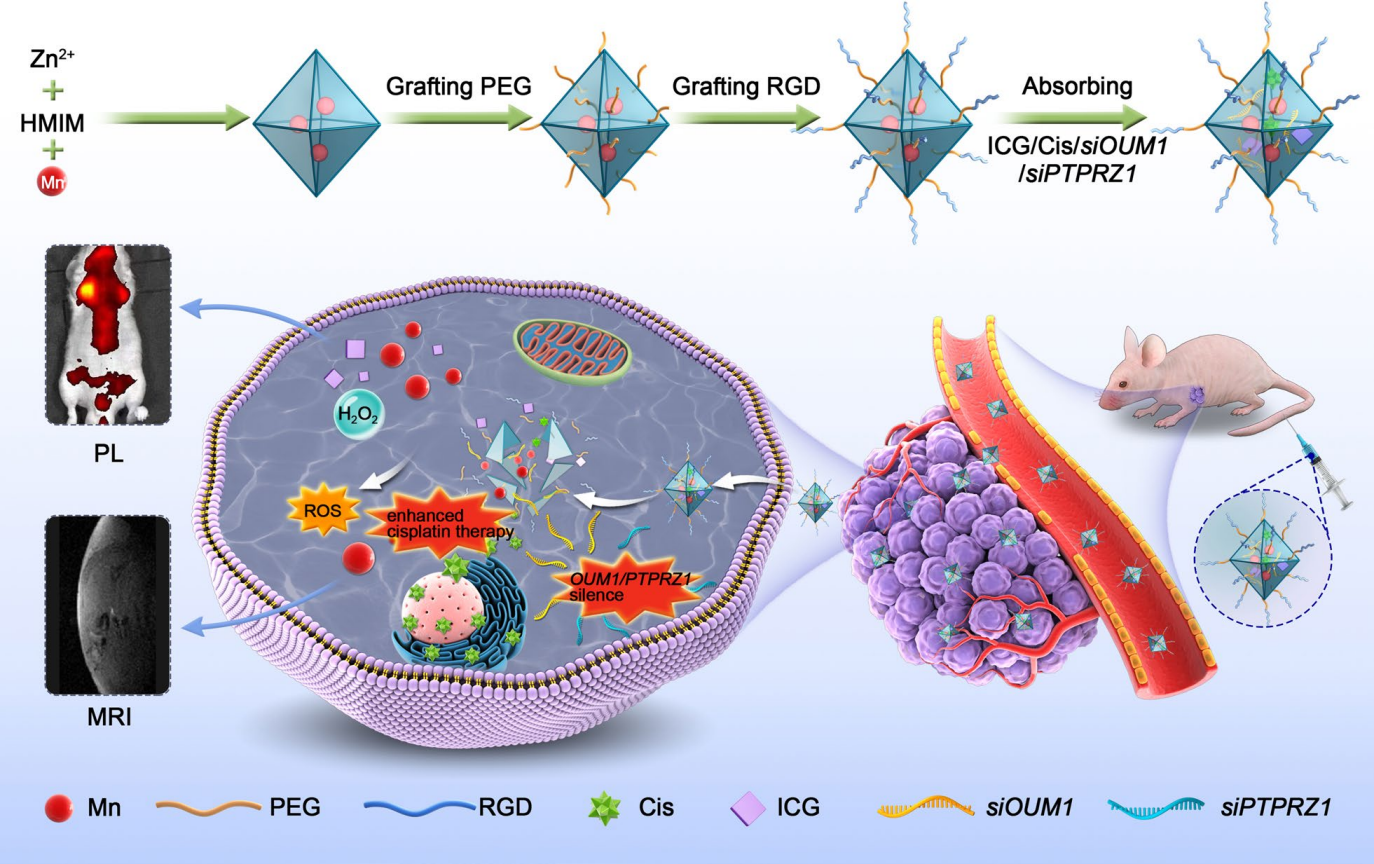
Schematic of key steps involved in preparation of porous ICG-COP@MOF-PR (ICG-*siOUM1* + *siPTPRZ1* + Cis@MOF-PR) nanostructures and their associated major mechanistic pathways in cancer therapy

**Fig. 5:**
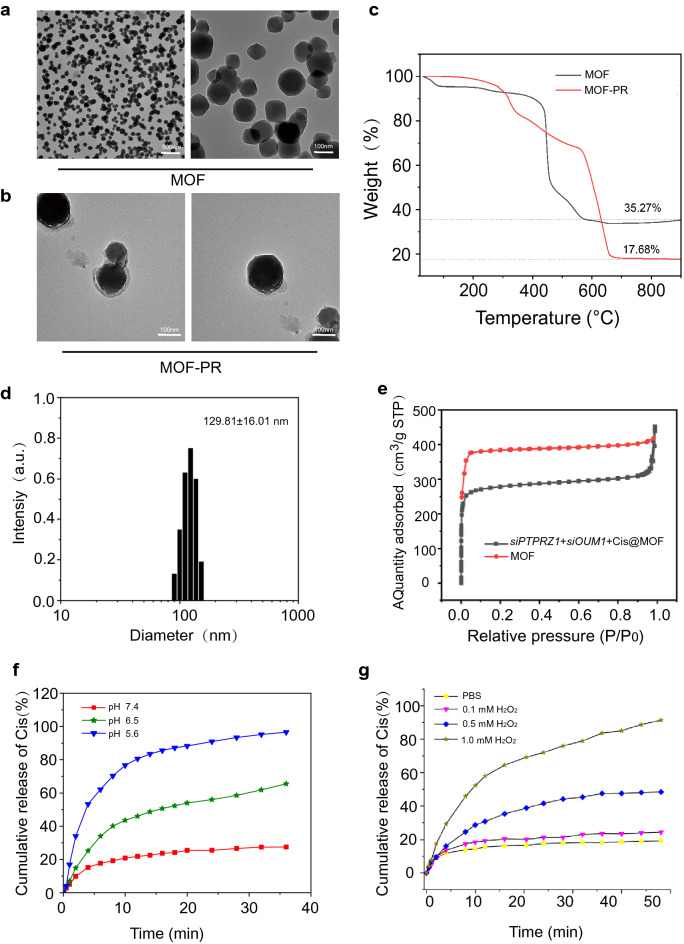
Synthesis and characterization of NPs. **a** TEM images of MOF. **b** TEM images of MOF-PR. **c** Thermogravimetric analysis of MOF and MOF-PR. **d** The NP size distribution of MOF-PR. **e** Nitrogen adsorption of MOF and *siOUM1* + *siPTPRZ1* + Cis@MOF. **f** Cisplatin release from Cis@MOF-PR in solutions with different pH values. **g** Cisplatin release from Cis@MOF-PR under acidic and H_2_O_2_ conditions

At present, RNA interference-mediated therapy has suffered from a lack of effective oligonucleotide-delivery systems. Traditional intravenous chemotherapy lacks specificity, and chemotherapy drugs also produce toxic and adverse side effects on normal tissues. To address the lack of effective oligonucleotide-delivery systems, drug delivery NPs based on Mn@MOF were designed to overcome the limitations of free therapeutics and to navigate biological barriers, as illustrated in Fig. 4. The MOF and MOF construction methods are described in the experimental section. The TEM images in Fig. 5a and Fig. 5b revealed the uniform polyhedron morphology of MOF and MOF-RGD-PEGTK (MOF-PR) within nanoscale dimensions. The MOF surface became rougher after PEG and RGD decoration, and the diameter of the NPs was increased. The MOF and MOF-PR showed ca. 62.64% and 81.98% mass loss, respectively, compared with the parent MOF (Fig. 5c), which indicates that approximately 19.34% RGD and PEG were absorbed into the MOF NPs. As determined by DLS, the hydrodynamic diameter of MOF-PR was 129.81 ± 16.01 nm (Fig. 5d). As illustrated in Fig. 5e, both exhibited a typical type I N_2_ absorption − desorption isotherm, which is considered one of the major characteristics of microporous materials. The BET surface area of the MOF was 1176 m^2^ g^−1^, whereas this value decreased to 563 m^2^ g^−1^ for *siPTPRZ1* + *siOUM1* + Cis@MOF. Furthermore, the corresponding pore volumes of *siPTPRZ1* + *siOUM1* + Cis@MOF and pure MOF nanostructures were 0.19 cm^3^ g^−1^ and 0.79 cm^3^ g^−1^, respectively. The sharp reduction in the BET surface area and pore volume demonstrated abundant drug molecules inside the MOF NPs. In this study, Zn^2+^ was an essential element in constructing the MOF and the structure prepared from zinc ion and 2-methylimidazolate is stable under nearly neutral aqueous environment but could be decomposed in acidic conditions [40]. This enabled the pH-responsive drug delivery characterization of the MOF. To explore the pH-responsiveness of the nanoparticles, the release profile of cisplatin from Cis@MOF-PR under pH 5.6, 6.5 and 7.4 was performed. As shown in Fig. 5f, a 40-min sustained release profile of cisplatin from Cis@MOF-PR NPs was observed. The amount of drug released by Cis@MOF-PR NPs was significantly higher under solution with lower pH. The drug released by the NPs was acidic sensitivity. Moreover, in this study, MOF was coated with TK linkers, which is ROS- responsive. When simulated with ROS, the ROS-responsive TK linkers would break and drugs loaded in the pores of MOF would release [41]. To further investigate the ROS-responsiveness of the nanoparticles, the release profile of cisplatin from Cis@MOF-PR was performed in phosphate buffered saline (PBS), H_2_O_2_ and acidic H_2_O_2_. As illustrated in Fig. 5g, a 60-min sustained release profile of cisplatin from Cis@MOF-PR NPs was observed. Approximately 90% of cisplatin was released from the nanovehicles within 48 min in acidic H_2_O_2_ with 1 mM H_2_O_2_, whereas in H_2_O_2_ and PBS, the amount was reduced to 54.0% and 29.3%, respectively, over the same duration. The H_2_O_2_, as well as acidity had a positive and significant impact on the release rate of cisplatin from Cis@MOF-PR NPs. These evidences demonstrated that the features of the drug released by the nanocarrier were ROS and acidic sensitivity.

### NPs could be taken up into UM cells to perform the cell-killing effect

**Fig. 6:**
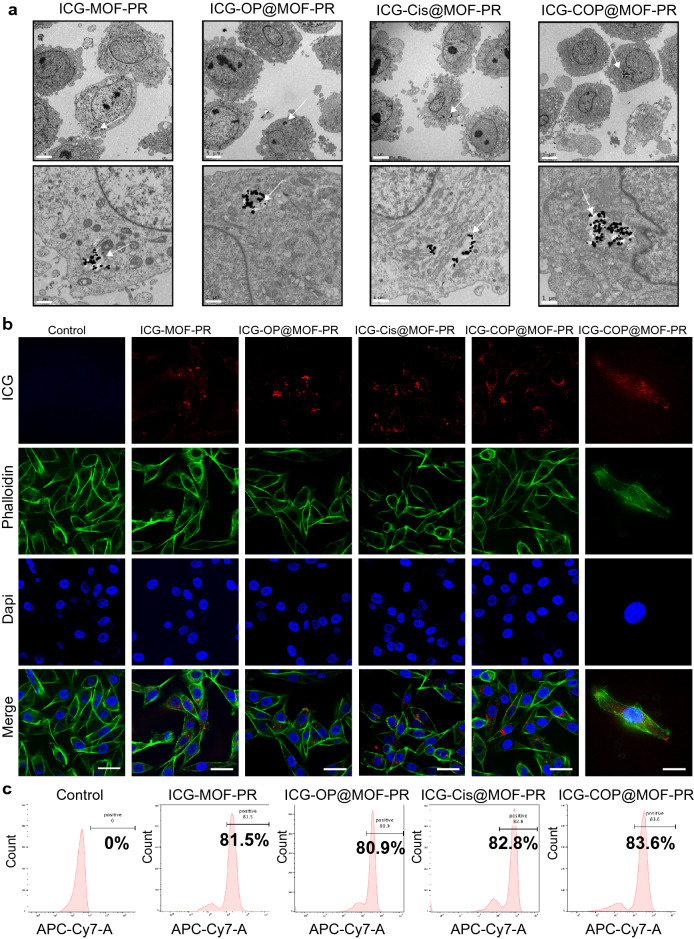
Cellular uptake of NPs. **a** TEM images indicate the intracellular distribution of NPs in OCM1a. Arrows: NPs uptaken into the cells. The scale bars in the first row and the second row are 5 μm and 1 μm, respectively. **b** From up to down, the CLSM images show ICG labeled NPs (red), cytoskeleton stained with FITC fluorescence-phalloidin (green), and cell nuclei stained with DAPI (blue) in OCM1a. The scale bars in the left 5 columns and the right 1 column are 50 μm and 20 μm, respectively. **c** Cellular uptake of NPs evaluated by flow cytometer. Experiments were repeated for three independent times

The cellular uptake and intracellular distribution of NPs are very important for their biological activities. To investigate the cellular uptake of NPs, OCM1a cells were incubated with ICG-MOF-PR, ICG-OP@MOF-PR (ICG-*siOUM1* + *siPTPRZ1*@MOF-PR), ICG-Cis@MOF-PR and ICG-COP@MOF-PR overnight at 37 ℃ and then the uptake of NPs was the investigated by TEM, which confirmed the incorporation of NPs into OCM1a cells (Fig. 6a). ICG in NPs serves as a fluorescence probe allowing for detection by CLSM and IVIS. OCM1a cells were then incubated with NPs overnight at 37 ℃, and the fluorescence intensity of ICG was then analyzed by CLSM. The results further confirmed the uptake of fluorescent NPs into OCM1a cells (Fig. 6b). The ICG fluorescence intensity of OCM1a cell-incubated NPs was also observed via a flow cytometer (Fig. 6c).

**Fig. 7:**
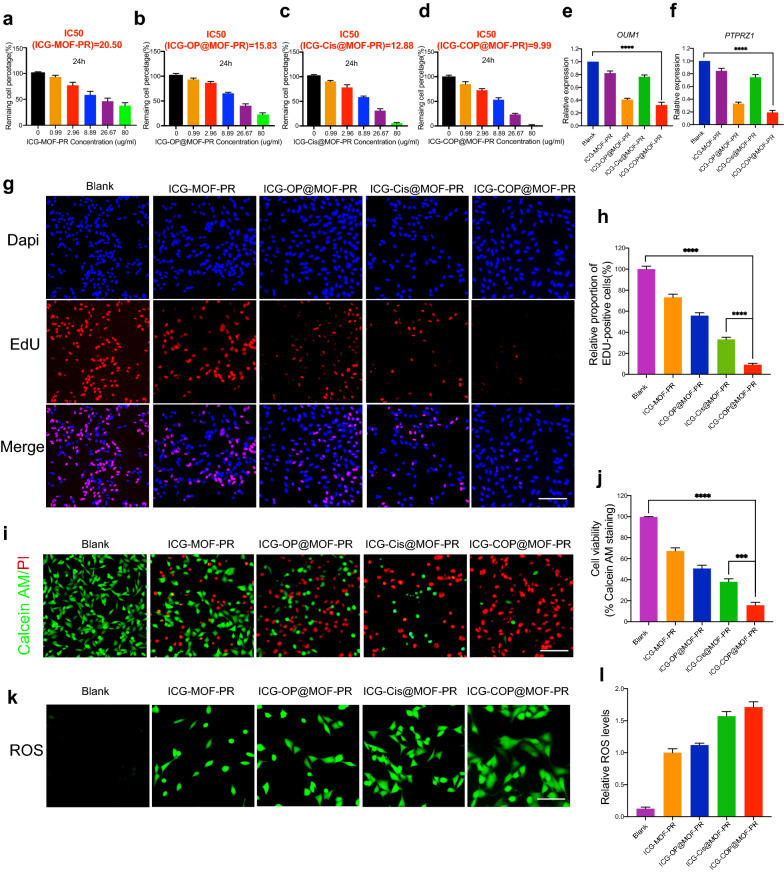
Biological activity of NPs. **a**–**d** Dose-dependent cell viability of OCM1a and IC50 values of NPs at 24 h. **e**, **f** Lnc *OUM1* and *PTPRZ1* expression in OCM1a after treated with NPs for 48 h. **g**, **h** EDU staining of OCM1a after treated with different NPs for 24 h. Scale bars, 200 μm. **i, j** Cell survival/death by live/dead staining (Calcein AM: live cells, PI: dead cells). Scale bars, 200 μm. **k**, **l** ROS production of OCM1a after treated with different NPs for 24 h. Scale bars, 100 μm. All data were obtained from at least three independent experiments (n ≥ 3), *** *P* < 0.001. **** *P* < 0.0001

Further studies were then conducted to evaluate the antitumor capacities of the NPs. First, CCK8 was employed to study cell viability. The results shown in Fig. 7a–d indicate dose-dependent cell viability. The IC_50_ values of ICG-MOF-PR, ICG-OP@MOF-PR, ICG-Cis@MOF-PR and ICG-COP@MOF-PR on OCM1a cells were 20.50, 15.83, 12.88 and 9.99 μg mL^−1^, respectively. The PCR results demonstrated that *OUM1* and *PTPRZ1* expression in OCM1a cells was significantly knocked down by ICG-OP@MOF-PR and ICG-COP@MOF-PR (Fig. 7e, f). EdU staining was then performed using OCM1a cells incubated with NPs for 24 h. The results revealed that the cell proliferation inhibition effect of ICG-COP@MOF-PR was greatly improved compared with that of ICG-Cis@MOF-PR (Fig. 7g, h). The CCK8 and EdU results were also echoed by live and dead staining of the cells after various treatments (Fig. 7i, j). Again, ICG-COP@MOF-PR exhibited the highest cell killing effect, whereas partial cell death was observed with ICG-OP@MOF-PR and ICG-Cis@MOF-PR. The MOF system constructed in our study degraded in the mildly acidic tumor microenvironment and released drugs loaded in the micropores and Mn^2+^, which induced a Fenton-like reaction and produced cytotoxic ROS to exert a cell killing effect. We then observed the ROS production induced by ICG-MOF-PR, ICG-OP@MOF-PR, ICG-Cis@MOF-PR and ICG-COP@MOF-PR. As shown in Fig. 7k, l, ROS were detected in OCM1a cells treated with these MOF NPs, which indicated the high CDT efficacy of this NP system. Taken together, the results indicate that ICG-COP@MOF-PR exhibited the best cell killing effect through a combination of ROS generation, selective siRNA knockdown and enhanced cisplatin treatment.

### In vivo analysis of the pharmacokinetics, targeting effect and dual-modal imaging capacity of intravenously injected NPs

Moreover, the pharmacokinetics of intravenously injected NPs were assessed to reveal the in vivo behaviors. At concentrations of 0, 5, 10, 15, and 20 mg kg^−1^, NPs were injected intravenously into Kunming mice. Initially, several major organs, including the heart, liver, lung, spleen, and kidney, were subjected to histological analysis after treatment with the indicated NP concentrations for 30 days. Hematoxylin and eosin staining of the abovementioned major organs in different groups showed no marked tissue abnormalities (Additional file 1: Fig. S6a). In addition, there was no significant difference in mouse body weights among the different groups (Additional file 1: Fig. S6b), further evidencing the excellent biocompatibility of NPs.

**Fig. 8:**
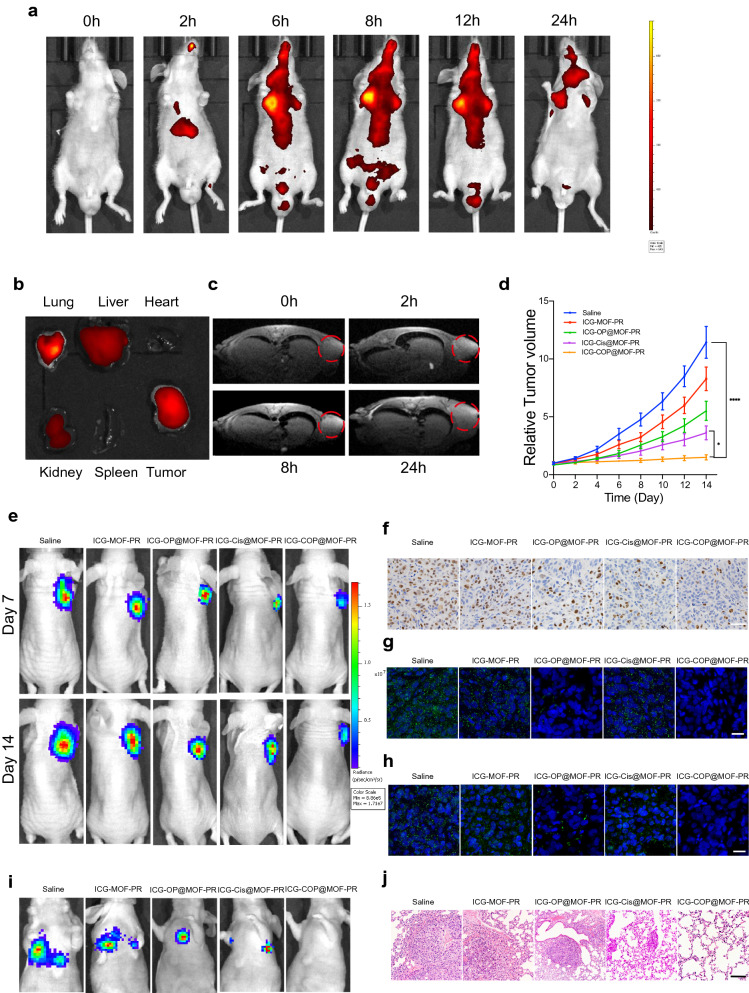
Pharmacokinetics and targeting effect and tumor suppression effects of intravenously injected NPs in vivo. **a** In vivo real-time bioluminescence imaging of UM tumor-bearing mice at different time points after administration of ICG-COP@MOF-PR. **b** ICG fluorescent intensities from ex vivo imaging of the major organs, and the tumors 24 h after injecting ICG-COP@MOF-PR. **c** T1-weighted MRI after intravenous injection with ICG-COP@MOF-PR at predesigned time points. **d**, **e** The tumor growth curves and bioluminescence imaging showed UM growth with different treatments in vivo. **f** Immunohistochemistry of Ki-67 of UM tumor samples with different treatments. Scale bar, 50 μm. **g** RNA in situ hybridization of lncRNA *OUM1* (green) in tumor samples with different treatments. Scale bar, 20 μm. **h** RNA in situ hybridization of *PTPRZ1* (green) in tumor samples with different treatments. Scale bar, 20 μm. **i** Bioluminescence imaging of M2b induced pulmonary metastasis models. **j** HE staining of metastasis nodes in the lung. Scale bar, 200 μm

A nanoplatform that supplies complementary anatomical and functional information about tumors by multimodal imaging is important for the precise detection and treatment of cancer. We therefore evaluated the multimodal imaging capacity of the multifunctional ICG-COP@MOF-PR nanoplatform in OCM1a-tumor-bearing mice. Because the fluorescence of ICG enabled its use as a desired agent for fluorescent imaging, we first evaluated the time-dependent biodistribution of ICG-COP@MOF-PR in OCM1a-tumor-bearing mice at predesigned time points (0, 2, 6, 8, 12 and 24 h) after the intravenous tail injection of NPs (Fig. 8a). We observed that fluorescence in the tumor region gradually increased over time and that the strongest fluorescence signal appeared at 8 h post-injection of ICG-COP@MOF-PR. We also discovered that fluorescence remained in the tumor region until 24 h post-injection, which suggested that ICG-COP@MOF-PR could accumulate in tumors for an extensive period. To better investigate the biodistribution of ICG-COP@MOF-PR, tumors and major organs (heart, liver, spleen, lung, and kidney) were collected after 24 h for ex vivo fluorescence imaging. As shown in Fig. 8b, the fluorescence signal was still present in the tumor tissue with partial liver, kidney and lung accumulation, which further confirmed the long retention period of NPs in vivo. Notably, the fluorescence imaging capability of ICG-COP@MOF-PR is beneficial to the real-time monitoring of its real-time in vivo distribution.

In the mildly acidic tumor microenvironment, the MOF in our nanodelivery system can be degraded, releasing Mn^2+^ and loaded drugs including cisplatin and siRNAs. The released Mn^2+^ not only facilitates CDT but will also affects the longitudinal relaxivity and MRI contrast effect, rendering the NPs a contrast agent for MRI [42]. As shown in Fig. 8c, ICG-COP@MOF-PR displayed an obvious and time-dependent T1-weighted enhancement in tumor tissues. Collectively, these data verified that ICG-COP@MOF-PR could be utilized as a safe versatile agent for fluorescence/MRI dual-modal imaging with high contrast, to precisely track their tumor accumulation and to provide more information about tumors from a more accurate and broader spectrum.

### Targeted siRNA interference-mediated therapy in synergy with CDT and enhanced cisplatin therapy suppressed UM tumor growth and pulmonary metastasis

To evaluate the therapeutic effect of NPs in vivo, a subcutaneous xenograft model constructed via subcutaneous injection of OCM1a cells and a pulmonary metastasis model induced through the intravenous tail injection of highly invasive UM cells, MUM2b cells were established. OCM1a-tumor-bearing BALB/c nude mice were randomly divided into five groups and then were treated with saline, ICG-MOF-PR, ICG-OP@MOF-PR, ICG-Cis@MOF-PR or ICG-COP@MOF-PR. The tumor sizes were measured every two days. After 14 days of treatment, significant inhibition and size reduction were observed in tumors treated with NPs (Fig. 8d). Notably, ICG-COP@MOF-PR showed superiority over ICG-Cis@MOF-PR in tumor inhibition and displayed the best tumor inhibition efficiency at all the investigated time points. Luciferase images of tumor-bearing mice at day 7 and day 14 also demonstrated the tumor inhibition effect of NPs (Fig. 8e). Tissue sections were prepared from tumor samples at 14 days after various treatments, and the expression levels of Ki-67 were evaluated by IHC staining. The treatments with ICG-MOF-PR, ICG-OP@MOF-PR, ICG-Cis@MOF-PR and ICG-COP@MOF-PR induced downregulation of Ki-67 expression, and among these treatments, ICG-COP@MOF-PR exerted the most significant suppression effect (Fig. 8f). RNA in situ hybridization of the lncRNAs *OUM1* and *PTPRZ1* was also performed with the abovementioned tumor samples to analyze the expression of the lncRNAs *OUM1* and *PTPRZ1* (Fig. 8g and h)*.* The sharp decrease in *OUM1* and *PTPRZ1* expression after ICG-COP@MOF-PR treatment demonstrated a high knockdown efficiency. This finding further confirmed that *siOUM1* and *siPTPRZ1* could be successfully loaded and transported into tumor cells and that *OUM1* and *PTPRZ1* were knocked down.

A pulmonary metastasis model was also evaluated by bioluminescence imaging and histopathological observation at day 14. The bioluminescence imaging approach is considered sufficiently sensitive and is able to detect as few as 100 tumor cells in vitro*,* and thus, tiny metastases that cannot be tracked by the naked eye can be captured [43]. As shown in Fig. 8i, all the mice in the saline group exhibited obvious metastases in the lung, whereas pulmonary metastasis was inhibited after treatment with NPs. In sharp contrast, pulmonary metastasis was significantly inhibited by treatment with ICG-COP@MOF with no detectable pulmonary metastases (Fig. 8i). Histologic micrographs (Fig. 8j) revealed the same results.

Taken together, the subcutaneous xenograft model and pulmonary metastasis model all suggested the superiority of ICG-COP@MOF-PR in inhibiting UM proliferation and metastasis. In our study, the release of cisplatin, *siOUM1* and *siPTPRZ1* exhibited ROS- and acid-sensitive characteristics, which indicates that the ROS generation in turn promoted the release of the abovementioned drugs. When ICG-COP@MOF-PR was taken up by UM cells, cisplatin, *siOUM1* and *siPTPRZ1* were released. Subsequently, *OUM1* and *PTPRZ1* could be selectively knocked down and protein tyrosine phosphorylation in the UM microenvironment was interrupted, leading to inhibition of proliferation and metastasis of UM. In addition, the knockdown of *OUM1* and *PTPRZ1* improved the sensitivity of UM cells to chemical drugs, enhancing the effect of cisplatin and further inhibiting UM proliferation and metastasis.

## Conclusion

In this study, we reported the identification of melanoma-specific lncRNAs and investigated the molecular and biological functions of *OUM1*. Microarrays and RACE assays confirmed a novel isoform of *LOC100505912* is a noncoding transcript in UM, and named *OUM1*. We clearly demonstrate that *PTPRZ1* contributes to tumor growth and metastasis as the downstream target of *OUM1* and is considered a novel oncogene in UM development. Here, RNA ChIP assays confirmed that *OUM1* mainly localized in the cytoplasm and functions by binding to PTPRZ1. In conclusion, we show that inhibition of the novel cytoplasmic lncRNA *OUM1* and its target gene *PTPRZ1* could suppress the proliferation and metastasis of UM in vivo and in vitro by interrupting protein tyrosine phosphorylation in the UM microenvironment.

Then, to overcome the limitations of the difficulty of drug administration and traditional therapeutics, a pH-responsive cisplatin, *siOUM1* and *siPTPRZ1* releasing manganese MOF with ICG as a fluorescent tag that degraded in the particular tumor microenvironment was fabricated. With a Fenton-like reaction induced by Mn^2+^, this NP system enabled the combination of CDT and siRNA interference-mediated therapy with enhanced cisplatin therapy, which greatly promoted treatment efficiency. In addition, with ICG as a fluorescence probe and Mn^2+^ as an outstanding contrast medium for MRI, this system was more precise in tracking and monitoring NP tumor accumulation and could provide more information about the tumor treatment process from a more accurate and broader spectrum. Furthermore, with manganese-enhanced MRI and ICG already applied in clinical practice, this multifunctional nanoplatform has high potential to be clinically translated to the clinical diagnosis and treatment of UM in the future.

## Materials and methods

### Patients and subjects

All samples were obtained from Shanghai Ninth Hospital. Informed consent for study participation was obtained from each patient under the authority of the Institutional Ethics Committee of Shanghai Ninth Hospital.

### Microarray and computational analysis

Briefly, RNA extracted from tissue samples (three melanomas and three normal tissues) was used to synthesize double-stranded cDNAs, which were labeled and hybridized to the 2.0 lncRNA Expression Microarray (Arraystar, Rockville, MD, USA). After hybridization and washing, the processed slides were scanned with the Axon GenePix 4000B microarray scanner (Molecular Devices, Sunnyvale, CA, USA). The raw data were extracted as paired-end read files using NimbleScan software (version 2.5; Roche NimbleGen, Inc., Madison, WI, USA), which performs quartile normalization and background correction of RNA. Differentially expressed genes were identified through the random variance model. The AP value was calculated using the paired t test. The thresholds set for up- and downregulated genes were fold change > 2.0 and P < 0.05. Hierarchical clustering was performed based on differentially expressed lncRNAs using Cluster_Treeview software from Stanford University (Palo Alto, CA, USA).

### Melanoma cell culture and siRNAs

The UM cell lines OM431, OCM1, C918, VUP, MUM2b and SP6.5 M were maintained in Dulbecco’s Modified Essential Medium (DMEM) (Gibco, Carlsbad, CA, USA) with 10% fetal bovine serum (FBS); OCM1a cells were maintained in Iscove’s Modified Dulbecco’s Medium (IMDM) (Gibco, Carlsbad, CA, USA) with 10% FBS; and control normal retinal pigment epithelial (RPE) cells were maintained in DMEM with 10% FBS. All the cells were cultured at 37 °C in an atmosphere with 5% CO_2_. Three different siRNAs targeting *OUM1*, two different siRNAs specifically targeting *PTPRZ1*, and a scrambled siRNA control were designed and synthesized (TUORAN, Shanghai, China) (Additional file 1: Table. S2). A total of 2 × 10^5^ cells were seeded into six-well plates. After 24 h, the cells were transfected with 150 nM siRNA using Lipofectamine 2000 (Invitrogen, Carlsbad, CA, USA) according to the manufacturer’s instructions. The transfection efficiency was optimized using a fluorescein-labeled negative control. After the indicated time, the cells were harvested for assays.

### ShRNA plasmid construction, lentivirus packaging, cloning and stable transfection

The shRNA sequence (5′-CATGAGTCTACCTACATAA-3′) targeting *OUM1* was obtained by PCR with primers containing Xho I and Mlu I sites and then cloned into the Xho I and Mlu I sites in the pGIPZ lentivirus vector (System Biosciences, CA, USA). 293 T cells were cultured in DMEM supplemented with 10% FBS at 37 °C; 6,000,000 of these cells were transfected with 3 µg of GIPZ-*shOUM1*, 3 µg of pMD 2. D and 6.0 µg of PsPax using Lipofectamine 2000 (Invitrogen, Carlsbad, CA, USA). After overnight incubation, the medium was replaced with 5 mL fresh medium. The virus-containing supernatants were collected at 48 h and 72 h after transfection, mixed and then filtered through a 0.45-μm cellulose acetate filter (Sartorius). The viral supernatants were concentrated with Amicon Ultra-15 Centrifugal Filter Units (Millipore, USA) at 4 °C and 5,000 rpm for 30 min. Selection was performed by incubation with 4 μg mL^−1^ puromycin for 2–4 weeks. To obtain stable cells, cultures were maintained in the presence of 2.5 μg mL^−1^ puromycin, and the expression of green fluorescent protein (GFP) was monitored using an inversion microscope. The transduction efficiency was determined based on GFP expression, and the knockdown efficiency was measured by qRT-PCR.

### Protein tyrosine phosphatase assay

Cells were maintained in 150 mm dishes, washed twice with PBS and lysed in lysis buffer (50 mM HEPES, pH 7.4, 0.5% Triton X-100, 10% glycerol, and 1 mM PMSF). A commercially available kit (Promega, Madison, WI, America) was used to measure cellular tyrosine phosphatase activity. Endogenous phosphate was removed using the provided spin columns according to the kit instructions. Subsequently, the protein concentration was determined using the BSA protein assay, and 40 µg of protein was aliquoted. Two chemically synthesized phosphopeptides, END(pY)INASL (P1) and DADE(pY)LIPQQG (P2), were used as substrates. The experiment was repeated at least three times. The phosphatase reaction mix was incubated for 45 min at 37 °C. An equal volume of the molybdate dye/additive mixture was added to all the wells for 15 min to stop the reactions. The phosphate concentration was determined at 630 nm with a plate reader.

### Preparation of NPs

The preparation of Mn@MOF (MOF) NPs was achieved using a previously reported scheme with minor adjustments [44]. Briefly, Mn (NO_3_)_2_ (20 mg) and HMIM (0.28 g) and Zn(NO_3_)_2_·6H_2_O (0.84 g) were dissolved in 10 mL DMF, placed in a glass tube and stirred well. The solution of the mixture was placed into a microwave reactor and heated to 130 °C for 5 min. The synthesized MOF NPs were obtained after centrifugation at 9,000 rpm for 20 min, and the precipitate was then washed three times with DMF. Mn@MOF (10 mg mL ^−1^, dissolved in deionized water) was preactivated with EDC/NHS (pH 5.6, 5 mg mL^−1^) for 2 h at 37 °C. The activated MOF NPs were mixed with the carboxyl-PEGTK- carboxyl aqueous solution (2 mg mL^−1^) and stirred at room temperature for 24 h to form an amide bond between the amino group in the MOF NPs and the carboxyl group in the carboxyl-PEGTK-carboxyl to synthesize Mn@MOF-PEGTK (Mn@MOF-P). The Mn@MOF-P was mixed with a carboxyl-PEG-NH_2_ aqueous solution (2 mg mL^−1^) and stirred at room temperature for 24 h to form an amide bond between the amino group in the RGD and the carboxyl group in the Mn@MOF-P for the synthesis of Mn@MOF-P-RGD (Mn@MOF-PR). The drug-loaded MOF was prepared by adding ICG and cisplatin, *siOUM1* + s*iPTPRZ1,* and *siOUM1* + s*iPTPRZ1* + Cis into the Mn@MOF-PR solution, and the drug was absorbed into the MOF through adsorption. For example, the Mn@MOF-PR NPs (10 mg mL^−1^), cisplatin (30 mg mL^−1^) and ICG (1 mg mL^−1^) in DMSO were mixed and stirred for 12 h. The ICG-Cis-loaded NPs were obtained after the mixture solution was centrifuged at 10,000 rpm for 10 min and washed three times with DMSO to remove the excess cisplatin. The same approach was employed for several other drug loadings.

### Characterization of NPs

The morphology of the NPs was observed by transmission electron microscopy (TEM, HT7700, Hitachi, Japan) images. The particle sizes were determined by dynamic light scattering (DLS). The chemical compositions of NPs were examined with Fourier transform infrared (FT-IR, Nicolet iS50, ThermoFisher Scientific, USA). Thermogravimetric analysis (TGA, TAG2, Mettler Toledo, Switzerland) was performed to evaluate the loading capacities of the NPs. The Brunauer–Emmett–Teller (BET) method was used performed to evaluate the surface areas of the NPs. The Cis@MOF-PR was soaked in solutions with different concentrations of H_2_O_2_ and different pH values. At each time point, the supernatant was collected, and an inductively coupled plasma emission spectrometer was utilized to test the concentration of Pt and thus determine the cisplatin leakage rate.

### Analysis of cellular uptake of NPs In Vitro (TEM, CLSM and flow cytometry)

A total of 1 × 10^6^ OCM1a cells were cultured in a T25 flask for 12 h. After aspirating the medium, the cells were incubated for 12 h with fresh medium containing 15 µg mL^−1^ ICG-MOF-PR, ICG-OP@MOF-PR, ICG-Cis@MOF-PR and ICG-COP@MOF-PR. The cells were washed twice with PBS, centrifuged and further fixed with 2.5% glutaraldehyde. Photos were taken by TEM ( HT7700, Hitachi, Japan). OCM1a cells (1 × 10^4^) were cultured in confocal laser scanning microscopy dishes for 12 h. After aspirating the medium, the cells were incubated for 12 h with fresh medium containing 15 µg mL^−1^ ICG-MOF-PR, ICG-OP@MOF-PR, ICG-Cis@MOF-PR and ICG-COP@MOF-PR. Subsequently, the cells were washed twice with PBS, fixed with 4% paraformaldehyde, washed twice, stained with DAPI and phalloidin, and observed using a CLSM (TCS SP8; Leica, Wetzlar, Germany). OCM1a cells (1 × 10^6^) were cultured in a T25 flask for 12 h. After aspirating the medium, the cells were incubated for 12 h with fresh medium containing 15 µg mL^−1^ ICG-MOF-PR, ICG-OP@MOF-PR, ICG-Cis@MOF-PR and ICG-COP@MOF-PR. The cells were digested, centrifuged and resuspended in PBS. The fluorescence was then detected via flow cytometry (BD FACS Calibur, BD Biosciences, CA, USA).

### Analysis of the effects of NPs on UM cell viability (CCK8 assay, EdU, Live/Dead and ROS measurement)

CCK8 assay: Cells were plated in 96-well plates (2000 cells/well in 100 µL), cultured overnight, and incubated with 0, 0.99, 2.96, 8.89, 26.67 and 80 μg mL^−1^ NPs for 24 h. Then, 10 µL of cell counting kit-8 (CCK-8, Dojindo, Japan) was added, and the cells were incubated at 37 °C in the dark for 4 h. The optical density at 450 nm was determined with a microplate reader. Live/dead, EdU, and ROS measurements: OCM1a cells were pretreated for 24 h with PBS, 15 µg mL^−1^ ICG-MOF-PR, ICG-OP@MOF-PR, ICG-Cis@MOF-PR and ICG-COP@MOF-PR. Cell survival (calcein AM staining) and death (PI staining) were assessed using a live/dead kit (Beyotime, China) according to recommended procedures. Cell proliferation was studied using a Cell-Light EdU Apollo488 In Vitro Kit (RiboBio, China) following the manufacturer’s instructions. The ROS levels were assessed using a DCFH-DA assay (Beyotime, China) according to recommended procedures. Images were taken with a fluorescence microscope (Nikon).

### The targeting effect of NPs

A subcutaneous xenograft model in nude mice was used to study the targeting effect of intravenously injected NPs in vivo. After tumor formation for 1 week, NPs were injected at a concentration of 15 mg kg^−1^ via the tail vein. The tumor and NPs were monitored with an IVIS at 0, 2, 6, 8, 12 and 24 h to verify the targeting effect of NPs in vivo. After 24 h, the mice were sacrificed and the heart, liver, spleen, pulmonary, kidney and tumor were removed for in vitro IVIS detection. The Animal Care and Use Committee at Shanghai Jiaotong Medical University approved the animal protocols.

### Antitumor and anti-metastasis efficacies of NPs

Luciferase-labeled OCM1a cells were subcutaneously injected into the right flanks of 4-week-old thymic nude mice. The mice were housed under a controlled environment in a sterile facility. After 1 week, the mice were randomly divided into 5 groups (n = 5) and treated with saline, ICG-MOF-PR, ICG-OP@MOF-PR, ICG-Cis@MOF-PR or ICG-COP@MOF-PR (saline and NPs were intravenously injected twice a week). The tumor volume was measured every 2 days with calipers. Tumor volume was calculated using the following formula: 0.5 × length × width × width. On days 7 and 14, an IVIS was used to detect the tumor. After 15 days, the mice were sacrificed, and the tumors were removed and analyzed. A pulmonary metastasis model was induced by the intravenous injection of 4-week-old thymic nude mice with luciferase-labeled MUM2b cells (1 × 10^6^ in 100 µL per injection) via the tail. Two weeks after cell injection, the mice were randomly divided into 5 groups (n = 5) and treated with saline, ICG-MOF-PR, ICG-OP@MOF-PR, ICG-Cis@MOF-PR or ICG-COP@MOF-PR (saline and NPs were intravenously injected twice a week). After two weeks of treatment, an IVIS was used for the evaluation of pulmonary metastasis. Subsequently, the mice were sacrificed, and the lungs were removed and analyzed. The Animal Care and Use Committee at Shanghai Jiaotong Medical University approved the animal protocols.

### Statistical analysis

All the statistical analyses were performed using SPSS version 19.0 (SPSS, Inc., Chicago, IL). For comparisons, one-way analysis of variance, independent samples t-tests and two-tailed Student’s t-tests were performed, as appropriate. Distribution and variance equality were analyzed for each gene in the melanoma and normal sample populations. For continuous variables, 2 different tests were used, Student’s t-test (for Gaussian populations with equal variance) and the Mann–Whitney test (for Gaussian populations with unequal variance), to determine the P value. P < 0.05 indicated statistical significance.

## Supplementary Information

**Additional file1: Fig. S1.** *OUM1* knockdown impairs UM cell metastasis and proliferation.** Fig. S2.** GO and pathway function analysis. **Fig. S3**. Bioinformatics analysis of *OUM1* targets. **Fig. S4.** *PTPRZ1* might be a target of *OUM1. Fig. S5. *Synergistic effect of SOV and *OUM1* interference on apoptosis. **Fig. S6. **In vivo toxicity evaluation. **Table S1. **Downregulated and Upregulated gene. **Table S2. **Primers and siRNA used in this study. **Table S3. **Primers used in RNA-ChIP assay. Details of the materials and methods of the *MTT assay, native PAGE, tumor xenograft model in nude mice, subcellular localization of OUM1, immunoblotting, IHC, RNA extraction, reverse transcription and qRT–PCR, cell migration assay, OUM1 DNA amplification and purification, RACE assay, genome-wide cDNA array*, *Gene Ontology (GO) analysis, RNA chromatin immunoprecipitation assay, survival index for SOV, soft agar assay, and colony formation assay based on paraffin-fluorescence probe-FISH*.

## Data Availability

The data that support the findings of this study are available from the corresponding author upon reasonable request.

## Abbreviations

UM: Uveal melanoma
PTP: Protein tyrosine phosphatase
PTPRZ1: PTP receptor type Z1
ICG: Indocyanine green
MOF: Metal–organic framework
RGD: Arginine-glycine-aspartate
NIR: Near-infrared region
MRI: Magnetic resonance imaging
PTKs: Protein tyrosine kinases
*OUM1*: UM Formation-Transcript 1
PEG: Polyethylene glycol
ROS: Reactive oxygen species
CDT: Chemodynamic therapy
NPs: Nanoparticles
CLSM: Confocal laser scanning microscopy
IVIS: in vivo Imaging system
IHC: Immunohistochemistry
qRT-PCR: Quantitative reverse transcription-polymerase chain reaction
RNA-ChIP: RNA-chromatin immunoprecipitation
SOV, V: Sodium orthovanadate
