# Supplementary material for: Targeted OUM1/PTPRZ1 silencing and synergetic CDT/enhanced chemical therapy toward uveal melanoma based on a dual-modal imaging-guided manganese metal–organic framework nanoparticles
Source: J Nanobiotechnology. 2022 Nov 5;20:472. doi: 10.1186/s12951-022-01643-y (PMC9636818; doi:10.1186/s12951-022-01643-y)
Supplement: Supplementary file 1 — Additional file1: Fig. S1. OUM1 knockdown impairs UM cell metastasis and proliferation. Fig. S2. GO and pathway function analysis. Fig. S3. Bioinformatics analysis of OUM1 targets. Fig. S4. PTPRZ1 might be a target of OUM1. Fig. S5. Synergistic effect of SOV and OUM1 interference on apoptosis. Fig. S6. In vivo toxicity evaluation. Table S1. Downregulated and Upregulated gene. Table S2. Primers and siRNA used in this study. Table S3. Primers used in RNA-ChIP assay. Details of the materials and methods of the MTT assay, native PAGE, tumor xenograft model in nude mice, subcellular localization of OUM1, immunoblotting, IHC, RNA extraction, reverse transcription and qRT–PCR, cell migration assay, OUM1 DNA amplification and purification, RACE assay, genome-wide cDNA array, Gene Ontology (GO) analysis, RNA chromatin immunoprecipitation assay, survival index for SOV, soft agar assay, and colony formation assay based on paraffin-fluorescence probe-FISH. [file 12951_2022_1643_MOESM1_ESM.docx]

**Supplementary information**

**Targeted *OUM1/PTPRZ*1 silencing and synergetic CDT/enhanced chemical therapy toward uveal melanoma based on a dual-modal imaging-guided manganese metal-organic framework nanoparticles**

Yue Li^1,2†^, Fang Li^1,2†^, Hui Pan^1,2^, Xiaolin Huang^1,2^, Jie Yu^1,2^, Xueru Liu^1,2^, Qinghao Zhang^4^, Caiwen Xiao^1,2*^, He Zhang^3*^, Leilei Zhang^1,2 *^

^1^ Department of Ophthalmology, Shanghai Ninth People’s Hospital afflicted to Shanghai Jiao Tong University School of Medicine, Shanghai 200011, China

^2^ Shanghai Key Laboratory of Orbital Diseases and Ocular Oncology, Shanghai 200011, China

^3^ School of Life Science and Technology, Tongji University, Shanghai 200092, China

^4^ East China University of Science and Technology, Shanghai 200237, China

^†^ These authors contributed equally to this work.

^*^ Corresponding authors.

E-mail addresses: [xiaocaiwen1855@shsmu.edu.cn(C](mailto:xiaocaiwen1855@shsmu.edu.cn(C). Xiao), [zhanghe@sjtu.edu.cn (H](mailto:zhanghe@sjtu.edu.cn%20(H). Zhang) [zhangleilei118001@shsmu.edu.cn](mailto:zhangleilei118001@shsmu.edu.cn) (L. Zhang).

**Fig. S1**. *OUM1* knockdown impairs UM cell metastasis and proliferation.

**a)** *LOC100505912* is described in detail. **b)** Schematic of *OUM1* and the siRNAs (S1, S2 and S3) used for the knockdown of *OUM1* expression. According to the NCBI databases, the lncRNA *OUM1* has a length of 898 bp, contains 4 exons, and is located at 4p15.2 (yellow box). The RACE assay detected a 1,464-bp transcript spanning 7 exons (blue box). Exons 5-7 were consistent with predicted exons 2-4, whereas exons 1 and 4 were located in the predicted 5’ UTR (blue box). **c-e)** *OUM1* expression decreased after *OUM1* silencing at 24 h (**c**), 48 h (**d**) and 72 h (**e**). **f)** MTT assays were used to detect the OCM1a cell proliferation rate after *OUM1* silencing. **g)** The metastasis rate was significantly reduced 48 h after *OUM1* silencing in OCM1a cells.

**Fig. S2.** GO and pathway function analysis.

The DNA chip results were analyzed to determine GO and pathway function differences between the stable *shOUM1* transfection group and the control group. Nucleotide binding was found to be involved and accounted for 32.1% after *OUM1* knockdown.

**Fig. S3**. Bioinformatics analysis of *OUM1* targets.

The bioinformatics analysis showed that nucleotide binding was related to transmembrane receptor protein phosphatases, which could affect PTP activity. PTP activity was significantly changed after *OUM1* knockdown.

**Fig. S4.** *PTPRZ1* might be a target of *OUM1.*

**a)** The *PTPRZ1* knockdown efficiency was detected by qRT-PCR and native PAGE after the treatment of OCM1a and OM431 cells with *siPTPRZ1*-1 and *siPTPRZ1*-2. **b)** Western blot analysis showed that *siPTPRZ1* efficiently silenced PTPRZ1 at the protein level in OCM1a cells. All experiments were performed 48 h following *siPTPRZ1* (125 pmol) or control siRNA (125 pmol) treatment. NC: nonsilencing control. **c)** qRT-PCR showed that *OUM1* expression was not significantly changed after the silencing of *PTPRZ1* in OCM1a and OM431 cells. **d-g)** Off-target analysis of *OUM1 ST8SIA1* **(d)** and *FXYD3* **(f)** Expression levels were detected by qRT-PCR in OCM1a*-shOUM1* and OM431-*shOUM1* cells; these transcripts were upregulated in UM cells but were downregulated after *OUM1* knockdown. *ST8SIA1* **(e)** and *FXYD3* **(g)** expression was determined to be similar in melanoma and normal tissues

**Fig. S5.** Synergistic effect of SOV and *OUM1* interference on apoptosis.

**a-d)** A panel of UM cells (MUM2b, VUP, SP6.5, and OCM1) was treated with SOV (10 μM, 25 μM, 50 μM, and 100 μM) for 24 h, 48, 72, and 96 h. Cell survival decreased with increases in the SOV concentration. **e, f)** OCM1a and OM431 cells were treated with increasing concentrations of SOV for 24, 48, 72 and 96 h. Cell viability was determined by MTT assays. Cell survival decreased significantly with an increase in SOV concentration. Approximately 99.8%, 82.0%, 57.7% and 12.7% OCM1a cell survival and approximately 98.0%, 81.8%, 30.1% and 5.1% OM431 cell survival were observed 96 h after treatment with SOV (10 μM, 25 μM, 50 μM, and 100 μM). **g, h)** Cell survival decreased significantly after *OUM1* knockdown in OCM1a and OM431 cells. Approximately 65.4%, 28.6%, 14.2% and 2.5% OCM1a cell survival and approximately 59.4%, 19.8%, 7.8% and 4.5% OM431 cell survival were detected 96 h after treatment with SOV (10 μM, 25 μM, 50 μM, and 100 μM).The error bars represent the± SD.

**Fig. S6.** In vivo toxicity evaluation.

**a)** Images of HE-stained sections obtained from the major organs (heart, liver, spleen, lung, and kidney) of mice 30 days after injection with ICG-COP@MOF-PR at various concentrations. **b)** Body weights of mice treated with ICG-COP@MOF-PR for 30 days. Scale bars, 100µm. All the data were obtained from at least three independent experiments (n≥3), P < 0.05. Each group contained 5 animals.

**Table S1.** Downregulated and Upregulated gene

| Style | Gene Symbol | Folds | Gene Symbol | Folds | Gene Symbol | Folds |
| --- | --- | --- | --- | --- | --- | --- |
| Down | MAMDC2 | 6.063876 | CD200 | 2.576953 | HIST1H2BK | 2.213201 |
| Down | GLDC | 5.86693 | SHANK2 | 2.501019 | SMC6 | 2.202729 |
| Down | SERPINA3 | 5.305334 | DDX5 | 2.487289 | SRSF11 | 2.200271 |
| Down | ITIH5 | 5.24152 | APOC2 | 2.485054 | EMX2 | 2.199951 |
| Down | PTPRZ1 | 3.966454 | CLDN11 | 2.471367 | SLC24A5 | 2.187565 |
| Down | PRSS23 | 3.785748 | ZNF114 | 2.426716 | GPNMB | 2.165953 |
| Down | SLC5A3 | 3.774021 | FBXL13 | 2.422084 | EXOC5 | 2.147204 |
| Down | LDB3 | 3.603171 | GOLGA8A | 2.399016 | ACYP2 | 2.138128 |
| Down | GAPDHS | 3.552752 | LCP2 | 2.397443 | FAM40B | 2.120427 |
| Down | FAM167B | 3.389602 | ANKRD36 | 2.396415 | RPS27A | 2.107895 |
| Down | TUBB7P | 3.381169 | ATP5G3 | 2.394336 | HMCN1 | 2.092241 |
| Down | MBP | 3.251386 | KCNAB1 | 2.39252 | LDB2 | 2.087717 |
| Down | FXYD3 | 3.240254 | S100A3 | 2.359915 | CSAD | 2.068797 |
| Down | COL7A1 | 3.080418 | ST8SIA1 | 2.342708 | PMP22 | 2.067203 |
| Down | RPS23 | 3.030498 | ITIH6 | 2.334517 | KRCC1 | 2.066464 |
| Down | C1orf63 | 3.00418 | VEPH1 | 2.328185 | KIAA1598 | 2.064431 |
| Down | EFCAB7 | 2.829186 | GPC6 | 2.274671 | HNRNPR | 2.055222 |
| Down | ART3 | 2.823898 | ZBTB1 | 2.274232 | EIF4A2 | 2.05407 |
| Down | PRDM7 | 2.807755 | ALDH1A1 | 2.265688 | MYEF2 | 2.048038 |
| Down | ITGB8 | 2.799134 | PCBP2 | 2.264885 | FBXO43 | 2.043541 |
| Down | EDIL3 | 2.796858 | LAMA4 | 2.26232 | VSTM2B | 2.043303 |
| Down | LPL | 2.793364 | HNRPDL | 2.261586 | BMP8B | 2.041128 |
| Down | SEMA5A | 2.790797 | PRKCB | 2.260097 | HNRNPU-AS1 | 2.034842 |
| Down | COL6A3 | 2.693469 | EVI2A | 2.255492 | EPHA3 | 2.021212 |
| Down | ITGB1BP1 | 2.661165 | PRPF4B | 2.25278 | RNF150 | 2.015424 |
| Down | MGP | 2.653783 | EIF5 | 2.232025 | CCNL1 | 2.01472 |
| Down | S100A4 | 2.643221 | CAV1 | 2.21744 |  |  |
| Up | VCX | 8.860259 | RNLS | 2.389676 | LIMCH1 | 2.148902 |
| Up | DSP | 4.927352 | HIST1H2BH | 2.370906 | CHRFAM7A | 2.140752 |
| Up | FSTL5 | 3.795521 | SPRY1 | 2.34207 | HSPA1A | 2.124477 |
| Up | ZSCAN18 | 3.608282 | PDGFC | 2.330443 | MTHFD1L | 2.097545 |
| Up | SERPINF1 | 3.59839 | KCTD12 | 2.318461 | CXADR | 2.084134 |
| Up | SCRG1 | 3.265927 | GDPD1 | 2.314813 | QKI | 2.08334 |
| Up | VEGFC | 3.10951 | NUS1 | 2.307616 | BNIP3 | 2.08025 |
| Up | CPE | 2.785378 | VEGFA | 2.29557 | EFNB2 | 2.063021 |
| Up | ARHGAP15 | 2.762142 | APOLD1 | 2.299487 | CPM | 2.061751 |
| Up | GPR160 | 2.648948 | PDK1 | 2.270782 | KIAA1804 | 2.058647 |
| Up | UCHL5 | 2.566051 | ELOVL7 | 2.251965 | ASNS | 2.056655 |
| Up | HRASLS | 2.562299 | SLC23A2 | 2.240914 | TPD52 | 2.052987 |
| Up | NOX4 | 2.547478 | MAGEA1 | 2.212241 | KLRG1 | 2.027294 |
| Up | ARMC9 | 2.532252 | ONECUT2 | 2.198349 | TGFBI | 2.018467 |
| Up | NFE2L3 | 2.509141 | SYNGR2 | 2.196614 | UBASH3B | 2.010459 |
| Up | CAP2 | 2.496675 | FOXC1 | 2.19229 | CYR61 | 2.008439 |
| Up | PRKAA2 | 2.473162 | SEPP1 | 2.162161 | RHOBTB1 | 2.008267 |
| Up | SLC16A6 | 2.44345 | MYO5B | 2.156541 | ERCC6 | 2.00558 |
| Up | NID1 | 2.418697 | S100A16 | 2.149939 | ELL2 | 2.002286 |
| Up | MMP1 | 2.788526 | TMEM200C | 2.299562 | CDS1 | 2.070499 |

| **Table S2.** Primers and siRNA used in this study | | | |
| --- | --- | --- | --- |
| Primer name | Sequence (5’- 3’) | Purpose |  |
| *OUM1*-F | TCCTTCAGCAGTGGTTTAAGGAAGT | qRT -PCR |  |
| *OUM1*-R | CGCTTTGAGAATTCCTAGTGTCATC | qRT -PCR |  |
| PTPRZ1-F | CAATCGCATAGGGACGAAATA | qRT -PCR |  |
| PTPRZ1-R | TAGTGACTGGTTGGGAAGTGG | qRT -PCR |  |
| FXYD3-F | TTCGGATAAACGCAGGACTC | qRT -PCR |  |
| FXYD3-R | AGGGTCACCTTCTGCATGTC | qRT -PCR |  |
| ST8SIA1-F | TGGCCCTTCTCTGTGAATATG | qRT -PCR |  |
| ST8SIA1-R | AAGTGGGCTGGAGTGAGGTAT | qRT -PCR |  |
| *OUM1*-siRNA-1-sense | CAUCUGUGCACGUUUGAGUdTdT | siLOC100505912 |  |
| *OUM1*-siRNA-1-antisense | ACUCAAACGUGCACAGAUGdTdT | siLOC100505912 |  |
| *OUM1*-siRNA-2-sense | CAUGAGUCUACCUACAUAAdTdT | siLOC100505912 |  |
| *OUM1*-siRNA-2-antisense | UUAUGUAGGUAGACUCAUGdTdT | siLOC100505912 |  |
| *OUM1*-siRNA-3-sense | GACCAUGCAGCCAAUUGUUdTdT | siLOC100505912 |  |
| *OUM1*-siRNA-3-antisense | AACAAUUGGCUGCAUGGUCdTdT | siLOC100505912 |  |
| PTPRZ1-siRNA-1-sense | CCCUUAUGCACCAACUAGAAA | siPTPRZ1 |  |
| PTPRZ1-siRNA-1-antisense | UCUAGUUGGUGCAUAAGGGUU | siPTPRZ1 |  |
| PTPRZ1-siRNA-2-sense | GUGGUUUCCUAGCUCUACAdTdT | siPTPRZ1 |  |
| PTPRZ1-siRNA-2-antisense | UGUAGAGCUAGGAAACCACdTdT | siPTPRZ1 |  |
| siRNA-Ctrl | ATCCACTACCGTTGTTATAGGTG | Negative ctrl siRNA |  |

| **Table S3.** Primers used in RNA-ChIP assay | | |
| --- | --- | --- |
|  | Primer name | Sequence (5’- 3’) |
| 1 | *OUM1*-F | CTGGGTATTTTTCTTTCATCTATTTTTCTTTCGGGCC |
|  | *OUM1*-R | CTCTGGTAGTTCATGAGCATGATGATTGGGT |
| 2 | *OUM1*-F | TCAGGTCAGATGCCCATTGTGC |
|  | *OUM1*-R | ACGTGCACAGATGAGGAGAGGT |
| 3 | *OUM1*-F | TGGAACCCTGGACCACTATGGA |
|  | *OUM1*-R | CAGCTCCAAGCCCTCAATCTTGTG |
| 4 | *OUM1*-F | GTCACTGACTCCTTCAGCAGTGG |
|  | *OUM1*-R | AGTGCAGCTTGTCTGTCCGTG |
| 5 | *OUM1*-F | GATGACACTAGGAATTCTCAAAGCGAGAG |
|  | *OUM1*-R | ACCAACCTGACCCTTCTGTAAGTTAGC |
| 6 | *OUM1*-F | GAGAACCTCAGCCTTCCAGAAGG |
|  | *OUM1*-R | TTTTTTTGGCTAGACTCAAAACTGATTACAATTAATCAATGT |

**Experimental Section**

*RACE assay:* Rapid amplification of cDNA ends (RACE) assays were performed as previously described. RACE-PCR was performed using the GeneRacer 5'/3' Kit (Invitrogen, Carlsbad, CA, USA), and PCR was performed using LA Taq DNA polymerase (Takara Bio, Otsu, Japan)

*Genome-wide cDNA array*: Total RNA was isolated from samples (untreated OCM1a cells and *shOUM1*-OCM1a cells) using an RNeasy Mini Kit (Qiagen, USA). cDNA was amplified, labeled and hybridized onto the mRNA Expression Microarray (Affymetrix PrimeView Human). The threshold set for up- and downregulated genes was fold change >2.0; the genes meeting this criterion were considered differentially regulated by the lncRNA *OUM1*. Hierarchical clustering was performed based on differentially expressed mRNAs. Genes were mapped onto KEGG pathways using DAVID version 6.7 (https://david.ncifcrf.gov/).

*Gene ontology (GO) analysis*: GO analysis was applied to analyze the main function of the differentially expressed genes according to GO, which is the key functional classification of the National Center for Biotechnology Information (NCBI). This analysis organizes genes into hierarchical categories and uncovers the gene regulatory network on the basis of biological process and molecular function.^1, 2^ Specifically, two-sided Fisher’s exact test and the χ^2^ test were used to classify the GO category, and the false discovery rate (FDR) ^3^ was calculated to correct the P value: the lower the FDR is, the smaller the error in judging the p value.

*RNA chromatin immunoprecipitation assay*: RNA chromatin immunoprecipitation (RNA ChIP) was conducted according to the manufacturer’s instructions (Millipore, CA, USA). Briefly, cells were fixed with 1% formaldehyde, and bound protein was digested with proteinase K. Immunoglobulin G was applied as a negative control. The primers utilized for amplifying *OUM1* are listed in Supplementary Table S3.

*Survival index for SOV*: UM cells were plated onto 96-well plates at a concentration of 1000 cells/well in triplicates. After an overnight incubation, the medium was changed to DMEM with 5% FBS, different concentrations of SOV were added, and the cells were incubated for an additional 24, 48, 72 or 96 h. Then, UM cell proliferation was determined using MTT assays as described previously.

*Soft agar assay*: soft agar assays were carried out in 6-well plates containing two agarose layers. The bottom layer contained 1.2% agarose, DMEM/IMDM and 20% FBS. After solidification of the bottom layer, the upper layer, which comprised 2 mL of 0.6% agarose, DMEM/IMDM with 20% FBS and the indicated cells (10,000 cells per well), was placed on top. The agarose layers were covered with 200 µl DMEM/IMDM supplemented with 10% FBS, which was refreshed every 3 days to avoid drying. Six-well plates were cultured for 2-3 weeks in a humidified incubator at 37 °C with 5% CO_2._ After 21 days of growth, the surviving colonies were fixed in methanol, washed two times with PBS, dried, stained with liquid crystal violet (Sigma-Aldrich, Dorset, USA), and counted.

*Colony formation assay*: Cells were seeded into 6-well plates at a density of 2×10^5^ cells/well, cultured for 24 h and then transfected with 150 nM siRNA. After transfection for 24 h, the cells were detached and collected. Cells from each treatment group (400 cells/well) were plated in duplicate in 6-well plates with DMEM or IMDM supplemented with 10% FBS. After incubation at 37 °C for 7 days, the cells were imaged. Then, the cells were fixed, stained with 0.1% crystal violet, and washed with 200 μL of 33% acetic acid. The absorbance of the collected liquid at 570 nm was measured with a microplate reader (Sigma-Aldrich, St. Louis, MO, USA).

*Paraffin-fluorescence* *probe-FISH*: The formalin-fixed, paraffin-embedded tissues were placed in the fixed fluid (diethyl pyrocarbonate, DEPC) for more than 12 h, dehydrated by gradient alcohol and embedded in paraffin. The paraffin was sliced using a slicer, and the slices were then heated in an oven at 62 °C for 2 h. Afterward, the slices were dewaxed, dehydrated and digested. Prehybridization solution was added to each section and incubated for 1 h at 37 °C. After removing the prehybridization solution, lncRNA *OUM1* or *PTPRZ1* probe hybridization was conducted and each section was incubated in a humidity chamber and hybridized overnight at 37 °C. The hybridization solution was then removed and washed, and the cell nuclei were stained with DAPI for 8 mins in the dark. The sections were visualized and imaged using a microscope (Nikon, Japan).

*Cell proliferation analysis (MTT assay):* Cells were plated in 96-well plates (2000 cells/well in 100 µL) and cultured overnight after transfection according to the manufacturer’s protocol, and 24, 48, 72, and 96 h after transfection, 10 µL of MTT solution (Sigma–Aldrich, St. Louis, MO, USA) was added. The cells were then incubated at 37 °C in the dark for 4 h. Subsequently, 150 mL of dimethylsulfoxide (DMSO) (Sigma–Aldrich, St. Louis, MO, USA) was added to solubilize the formazan crystals. The optical density at 490 nm was determined with a microplate reader.

*Native PAGE*: Equal amounts of real-time PCR products were separated by 10% native polyacrylamide gel electrophoresis (native PAGE). The gels were stained with SYBR green (Invitrogen, Carlsbad, CA) for 30 min, and the bands were visualized. The housekeeping gene GAPDH was used for normalization.

*Tumor xenograft model in nude mice*: Stable *shOUM1*-OCM1a cells and control OCM1a cells (1×10^6^ in 100 µL per injection) were subcutaneously injected into the right flanks of 4-week-old thymic nude mice. The mice were housed under a controlled environment in a sterile facility. The tumor volume was measured every 3-4 days with calipers. The tumor volume was calculated using the following formula: 0.5×length×width×width. After 31 days, the mice were sacrificed, and the tumors were removed and analyzed. The Animal Care and Use Committee at Shanghai Jiaotong Medical University approved the animal protocols.

*Subcellular localization*: Nuclear and cytoplasmic RNAs were purified using Thermo Fisher BioReagents (Thermo Fisher, USA) according to the manufacturer’s instructions. RT-PCR was performed as follows: 1 μL 3× Klen-Taq I Mix, 1 μL cDNA, and 0.5 μL of each primer at 10 μM were combined under liquid wax. After incubation at 95 °C for 2 min, cDNA was amplified through 30 cycles of denaturation at 95 °C for 30 sec and extension at 72 °C for 30 sec, followed by a final extension at 72 °C for 7 min.

*Western blot (immunoblot) analysis*: The protein extracted from cells was collected by centrifugation at 12,000 *g* at 4 °C for 30 min. The total protein concentration was determined using the BCA Protein Assay Kit (Pierce, Rockford, IL, USA). Equal amounts of total cellular extracts (20 µg) were separated by SDS-PAGE and blotted onto PVDF membranes (Millipore, USA). After incubation with blocking solution (5% nonfat dry milk/0.1% Tween-20/PBS), the membranes were incubated overnight at 4 °C with the recommended dilution of the following primary antibodies: anti-PTPRZ1 (1:1,000, BD, USA) and anti-ß-actin (1:5,000, Invitrogen, USA). Then, the membranes were washed three times with TBS containing 0.1% Tween-20 (TBST) and incubated with a secondary antibody (1:5,000, Invitrogen, USA) conjugated to a fluorescent tag for 1 h. The membranes were then washed three times with TBST. The bands were detected using the Odyssey Infrared imaging system (Odyssey; LI-COR, Lincoln, NE, USA).

*MR imaging*: For in vivo MR imaging, a subcutaneous xenograft model in nude mice was used. OCM1a cells were subcutaneously injected into the right flanks of 4-week-old nude mice. When the tumor size reached about 10 mm, NPs were injected at a concentration of 15 mg kg^−1^ via the tail vein. The tumors were monitored with small animal MR at 0, 2, 8 and 24h. Small animal MR images were collected and analyzed on an MR imaging scanner equipped with a special animal imaging coil. The Animal Care and Use Committee at Shanghai Jiaotong Medical University approved the animal protocols.

*Immunohistochemistry*: The specimens obtained from patients undergoing surgery or from tumor-bearing nude mice were cut into two pieces and then fixed with formalin for 24 h. The next day, the formalin-fixed tissues were embedded in paraffin to generate formalin-fixed, paraffin-embedded (FFPE) tissues, which were cut into 5-μm-thick sections. The tissue sections were washed with PBS and incubated with blocking buffer (PBS containing 10% normal goat serum, Invitrogen), 0.3% Triton X-100 (Sigma-Aldrich, USA) and 0.1% NaN_3_ (Sigma-Aldrich, USA) for 30 min. The coverslipped tissues were then incubated with primary antibodies against PTPRZ1 (1:200, BD, USA) and Ki-67 (1:200, BD, USA) at 4 °C overnight. The immunoreactive sections were visualized and imaged using a microscope (Olympus BX51, Japan).

*RNA extraction, reverse transcription and quantitative polymerase chain reaction*: Total RNA was isolated from UM cells and tissues using Trizol reagent (Invitrogen, Carlsbad, CA, USA) following the manufacturer’s protocol. cDNA was synthesized according to the manufacturer's protocol (Takara Bio, Otsu, Japan). Dpn1 (Sangon Biotech, Shanghai, China) was used to remove genomic DNA from the extracted RNA, which was used to amplify the lncRNAs. Screening for lncRNAs was performed by qRT-PCR with the primer sets described in Supplementary Table S1. PCR was performed according to the manufacturer's protocol (Takara Bio, Otsu, Japan) and was repeated at least three times for each sample. The target gene expression levels were normalized to those of GAPDH.

*Reverse transcription of OUM1 DNA:* Transcription reactions were performed in a thermal cycler in a total volume of 20 µL containing 2 μL of enzyme mix, 10 μL of 2× NTP/CAP, 1 μg of linear OUM1 template DNA, 0.5 μL of KAPA HiFi DNA polymerase (1 U μL^-1^), 2 μL of 10× reaction buffer, and ddH_2_O (mMESSAGE mMACHINE® Kit). After thorough mixing, the reaction was incubated for 2 h. Subsequently, 1 μL of TURBO DNase was added for 2 h to remove the template DNA. The concentration of OUM1 RNA was determined with a NanoDrop 2000.

*Cell migration assay*: Cells were seeded into 6-well plates at a density of 2×10^5^ cells/well, cultured for 24 h and then transfected with 150 nM siRNA. After transfection for 24 h, the cells were detached and collected. A total of 1×10^5^ cells from each treatment group were seeded in DMEM or IMDM supplemented with 1% FBS in an upper chamber containing a membrane with 8-μm pores (Millipore, Schwalbach, Germany); the lower chamber contained 1 mL of the corresponding culture medium supplemented with 20% FBS. After incubation at 37 °C for 72 h, the cells that migrated to the lower chamber were washed with PBS, fixed with 5% paraformaldehyde and stained with 0.1% crystal violet. The crystal violet was collected from the migrated cells using 200 μL of 33% acetic acid, and the absorbance of the collected liquid was measured at 570 nm with a microplate reader (Sigma-Aldrich, St. Louis, MO, USA). OCM1a and OM431 cells with stable shRNA expression were also treated as previously described.

*OUM1 DNA amplification and purification*: The *OUM1* overexpression plasmid was used as a template. The following primers were used for the amplification of *OUM1* DNA: sense: 5'-TAATACGACTCACTATAGGTTTTTTTTTTTTTTTAGC-3'; and antisense: 5'-GACCTCTCCTCGTCTGCACGGATGAGTGCCAACTC-3'. PCR was performed in a thermal cycler in a total volume of 25 µl in 8 tubes, each containing 100 ng genomic DNA, 5 picomoles of each primer, 0.5 μl KOD-Plus-Neo enzyme (1 U μl^-1^) (TOYOBO, Osaka, Japan), 5 μl 5× KAPA HiFi Buffer, and ddH2O to 25 µl. The PCR cycling conditions included an initial denaturation at 95 °C for 30 sec, followed by 40 cycles of denaturation at 95 °C for 5 sec, annealing at 55 °C for 20 sec, and extension at 72 °C for 50 sec, with a final extension step at 72 °C for 20 sec. The presence of a specific PCR product was confirmed using 1.5% agarose gel electrophoresis. The DNA AxyPrep Gel Extraction Kit was used to purify *OUM1* DNA according to the manufacturer’s instructions.

**References**

(1) The Gene Ontology (GO) project in 2006. *Nucleic Acids Res* **2006**, *34* (Database issue), D322-326. DOI: 34/suppl_1/D322 [pii]

10.1093/nar/gkj021.

(2) Ashburner, M.; Ball, C. A.; Blake, J. A.; Botstein, D.; Butler, H.; Cherry, J. M.; Davis, A. P.; Dolinski, K.; Dwight, S. S.; Eppig, J. T.; et al. Gene ontology: tool for the unification of biology. The Gene Ontology Consortium. *Nat Genet* **2000**, *25* (1), 25-29. DOI: 10.1038/75556.

(3) Dupuy, D.; Bertin, N.; Hidalgo, C. A.; Venkatesan, K.; Tu, D.; Lee, D.; Rosenberg, J.; Svrzikapa, N.; Blanc, A.; Carnec, A.; et al. Genome-scale analysis of in vivo spatiotemporal promoter activity in Caenorhabditis elegans. *Nat Biotechnol* **2007**, *25* (6), 663-668. DOI: nbt1305 [pii]

10.1038/nbt1305.
